# *Streptococcus suis* avian expansion suggests shared antibiotic use drives host jumps

**DOI:** 10.1186/s12915-025-02477-4

**Published:** 2025-12-02

**Authors:** Muriel Dresen, Gemma G. R. Murray, Peter Valentin-Weigand, Marcus Fulde, Lucy A. Weinert

**Affiliations:** 1 Department of Veterinary Medicine, Institute of Microbiology and Epizootics, Freie Universität Berlin, Berlin, Germany; 2 Department of Veterinary Medicine, University of Cambridge, Cambridge, UK; 3 Department of Genetics, Evolution and Environment, University College London, London, UK; 4 Institute for Microbiology, University of Veterinary Medicine Hannover, Hannover, Germany

**Keywords:** Comparative genomics, One Health, Host range

## Abstract

**Background:**

The interconnectedness of human, animal, and environmental health drives emerging threats, such as antimicrobial-resistant pathogens. The widespread use of the same antimicrobials in both human and livestock may play a role in interspecies bacterial transmission by disrupting natural microbial communities and creating an environment favouring resistant bacteria**.** Pigs and poultry receive high levels of antimicrobials and are reservoirs of multidrug-resistant bacteria, including *Streptococcus suis*, a zoonotic pig pathogen. *S. suis* detection in non-porcine hosts, particularly poultry, raises a critical question: is this due to transient spillover or does it represent sustained host jumps and adaptation?

**Results:**

Analysing over 3000 *S. suis* genomes from a diverse range of hosts—including pigs, wild boar, humans, cats, dogs, cattle, fish, otter, and birds—we identify a multidrug-resistant lineage, distinct from the lineage responsible for most zoonoses, that has undergone multiple host jump events into birds. Unlike transmission to humans, which is exclusively derived through contacts with pigs, we find evidence of *S. suis* adaptation to birds. This includes phylogenetic persistence, independent acquisition of bird-specific mobile genomic islands, enhanced survival in chicken versus pig blood, and subsequent transmission from poultry to wild birds.

**Conclusions:**

While chickens may not be a source of zoonotic *S. suis* infections, shared antibiotic usage in pigs and poultry may have promoted host jumps of multidrug-resistant *S. suis*, leading to onward transmission to wild bird populations. Our results suggest that antibiotic use in livestock production may promote transmission of antimicrobial-resistant bacteria to other hosts, thereby expanding the ecological range of bacterial pathogens.

**Supplementary Information:**

The online version contains supplementary material available at 10.1186/s12915-025-02477-4.

## Background

Antimicrobial resistance (AMR) is one of the biggest threats to modern medicine. In 2019 nearly five million deaths were associated with bacterial AMR, representing a huge economic burden to the healthcare sector, which is estimated to increase [1]. The simultaneous rise of AMR bacteria in humans, animals and the environment demonstrates their interconnectedness and the importance of a One Health approach to tackling this threat [2]. The widespread use of antimicrobials from the same class in human and veterinary medicine directly promotes the emergence of drug-resistant bacteria. Beyond this, these antibiotics decimate the natural microbiota within hosts. Such a reduction in microbial diversity leads to homogenised microbial communities [3]. These homogenised microbial communities are more prone to invasion of new pathogens—especially resistant ones [4]. Livestock animals, such as pigs and poultry, are especially at risk as they are exposed to the greatest volumes of antibiotics [5, 6] and frequently carry AMR bacteria [7]. Knowledge on whether or how these resistant bacteria are transmitted between various host species, especially among different farm animals, remains scarce.

One multidrug-resistant bacterium of pigs is *Streptococcus suis* [8], which commonly colonises the upper respiratory tract and is ubiquitous on pig farms worldwide [9, 10]. *S. suis* can also become invasive, causing severe disease with symptoms including bronchopneumonia, arthritis, pericarditis, meningitis, and sepsis, and is responsible for high economic losses in the pig industry [10, 11]. Furthermore, *S. suis* plays a substantial role as a zoonotic pathogen; it can cause a streptococcal toxic shock-like syndrome in humans and is the leading cause of adult meningitis in parts of South East Asia [12, 13]. Control of *S. suis* is compromised by substantial genetic diversity of the pathogen. This is reflected in the presence of more than 700 sequence types determined by multi-locus sequence typing (MLST) and 29 described serotypes based on *S. suis* capsular polysaccharides [14, 15]. However, Murray et al. showed that a small number of genetic lineages of *S. suis* are responsible for most cases of disease and zoonoses and they expanded alongside the intensification of pig farming [16]. The majority of porcine and human disease is caused by serotype 2 isolates [10]. However, infections in swine are also caused by other serotypes, such as serotype 9 [10, 17]. The capsule, the muraminidase-released protein (MRP), the extracellular factor (EF, *epf*) and the pore-forming toxin suilysin (SLY) have been described as important virulence and virulence-associated factors of *S. suis* [18].

The natural reservoirs of *S. suis* are pigs and wild boar, but it has also been isolated from many other host species such as cats, dogs, horses, sheep, cattle, wild rabbits, sea otter, fish and several bird species [19–30]; see Additional File 1: Table S1 for a summary. The presence of *S. suis* in multiple host species and its ubiquitous presence in farmed pigs where it is frequently exposed to antibiotic treatment, make it an excellent pathogen to study links between AMR and host transmission. In addition, *S. suis* is regarded an important reservoir for AMR genes and mobile genetic elements (MGEs) [31, 32]. Maintenance in different host species can be important in determining the evolutionary trajectory of a pathogen because different host environments can promote genetic diversity through differing selection pressures and gene exchange with host microbiomes [33]. This process may contribute to the evolution of new resistant or virulent lineages. Other host species may also play a role in maintenance of pathogens in pig populations through reseeding of infection [34]. However, whether non-porcine animal species play a role in the evolution and maintenance of *S. suis* in pigs is an open question.

As in pigs, in other farmed animals, *S. suis* can be both carried asymptomatically and cause invasive disease. In sheep and cattle, symptoms associated with *S. suis* include endocarditis, meningitis, pneumonia, and sepsis [27, 29, 35]. *S. suis* was also found in faeces of healthy cattle [36] and in one study, 67% cattle isolates were multidrug-resistant [27]. In the fish *T. pectoralis*, researchers have isolated *S. suis* as the causative agent of exophthalmos, haemorrhages and swimming disorders in a commercial fish farm [23]. In chickens, evidence has been found that *S. suis* is being carried by healthy birds and persisting independently of pigs. Nhung et al. examined carriage of *S. suis* in healthy chicken flocks, including pigs from the same or near-by farms in Vietnam [25]. They found that the commensal prevalence of *S. suis* in chickens is similar in farms raising either chickens only or chickens and pigs together. Chicken isolates were genetically distinct from pigs and had lower diversity. Moreover, 95% of the chicken isolates were multidrug-resistant compared to 87% of pigs [25]. There is no evidence of *S. suis* disease in chickens in the literature.

Like farmed animals, *S. suis* can also act as a commensal or an invasive pathogen in companion animals. *S. suis* has been isolated from tonsils and anal swabs of healthy cats and dogs [30], from cases of endomyocarditis and meningoencephalitis in cats [37, 38], and a case of a diseased dog with lethargy and polyuria [39]. Another study by Devriese et al. describes several case reports of *S. suis* isolated from different diseased bird species including psittacine birds, zebra finches, bullfinches, canaries, and a domestic duck [22]. Both the septicaemic infection and the pure *S. suis* cultures isolated from these birds resemble the pathogenesis in mammalian infections. Most of these isolates belong to serotype 9 [22].

Finally, *S. suis* has also been found in wild animals. Wild boars are considered a natural reservoir host for *S. suis* and are often asymptomatic carriers [19]. However, wild boar isolates have been found to also include serotype 2 isolates positive for the virulence factors MRP, EPF and SLY which pose a potential risk for zoonotic infection [19, 24, 40]. *S. suis* has also been isolated from wild rabbits in Spain. Most of these isolates were assigned to serotype 9 [41]. The *S. suis* isolate found in a Eurasian river otter belongs to serotype 2 and is positive for the virulence factors hyaluronidase (Hyl), arginine deiminase (ArcA) and protective antigen (Bay046) but negative for MRP, SLY and EF [26].

It is generally assumed that, as in humans, infections in non-porcine hosts are due to “spillovers” from pigs instead of adaptation to other animal hosts [26, 39, 42]. However, there are many infections with no known pig contact and there has been no large-scale genomic investigation of host specificity across *S. suis* [37, 43]. Moreover, it is not known whether *S. suis* persists in these animal species and whether they represent a potential infection source for pigs or humans. To investigate this and potential host adaptation mechanisms and drivers, we collected 22 publicly available genomes of *S. suis* from atypical host species, sequenced 17 additional isolates from Germany (Additional File 2: Figure S1, Additional File 1: Table S2) and contextualised these with more than 3000 isolates from pigs, wild boars and humans [16]. We used phylogenetic analyses to investigate host adaptation as phylogenetic clustering is a hallmark of persistence and transmission among animal species [44]. As isolates from chicken and cattle have previously been shown to be multidrug-resistant [25, 27], we screened for antimicrobial resistance genes in our animal isolates. Applying BEAST analysis, a tool for evolutionary phylogenetic analysis [45], we find evidence of multiple jumps of a multidrug-resistant lineage of *S. suis* from pigs to different bird species. Bird isolates contain mobile genetic elements not found in pigs, along with a trend for enhanced survival in bird blood. Given that we only observe these jumps from a multidrug-resistant group of isolates, we suggest the shared use of antibiotics may have facilitated these host jumps from pigs and the persistence of *S. suis* in poultry and birds. The presence of *S. suis* in other animal species was not linked to AMR and did not show signs of host adaptation.

## Results

### S. suis isolates from non-pig hosts mostly belong to the commensal group

To extend our collection of *S. suis* isolates from atypical hosts sampled in Germany, we combined our isolates with publicly available *S. suis* isolates from different animal species. We found published sequences of 20 chicken isolates from Vietnam [25], one cattle (USA), one lamb (Canada), one cat (Malaysia) [46], one fish (Thailand) [23] and one otter isolate (South Korea) [26]. All publicly available sequences included in this study are summarised in Additional File 1: Table S3. Our *S. suis *in silico serotyping of the new isolates [47] revealed that while these isolates had a range of serotypes, the most common was serotype 9 (14/37 non-pig hosts). All isolates were negative for the three virulence markers *mrp*, *epf* and *sly*, with the exception of the otter isolates that contained the *mrp* gene (although previously reported negative [26]). All isolates belonged to novel multi-locus sequence types (MLST) except for the otter isolate (MLST type 28) (Table 1). Genome clustering with PopPUNK [48] revealed that except for the otter isolate (pathogenic lineage 2/ST28), all other isolates belonged to previously undescribed lineages, with most of the bird isolates from newly characterised lineages 590, 592, 594, 595 and 599. In total, 16 new lineages that consisted exclusively of avian isolates were identified (Table 1). Fifteen of the avian and one dog isolate formed the new clonal complex (CC) 4014. The otter belonged to CC28 and all the other new isolates formed singletons (Additional File 2: Figure S2 [49–51], Additional File 1: Table S4).

**Table 1:** Serotypes and PopPUNK lineages of *S. suis* isolates used in this study

Isolate	Host species	Country of origin	Serotype	PopPUNK Lineage
Otter	*Lutra lutra*	South Korea	2	2
3504_Bird	*Gallus gallus*	Vietnam	Non-typeable	**590**
3509_Bird	*Gallus gallus*	Vietnam	Non-typeable	
3528_Bird	*Gallus gallus*	Vietnam	Non-typeable	
3537_Bird	*Gallus gallus*	Vietnam	Non-typeable	
3541_Bird	*Gallus gallus*	Vietnam	Non-typeable	
3550_Bird	*Gallus gallus*	Vietnam	Non-typeable	
3559_Bird	*Gallus gallus*	Vietnam	Non-typeable	
3539_Bird	*Gallus gallus*	Vietnam	Non-typeable	**592**
3544_Bird	*Gallus gallus*	Vietnam	Non-typeable	
3546_Bird	*Gallus gallus*	Vietnam	Non-typeable	
3519_Vietnam_Pig	*Sus scrofa*	Vietnam	Non-typeable	**593**
3521_Vietnam_Pig	*Sus scrofa*	Vietnam	Non-typeable	
3523_Vietnam_Pig	*Sus scrofa*	Vietnam	Non-typeable	
IMT52389_Bird	*Melopsittacus undulatus*	Germany	Non-typeable	**594**
IMT52716_Bird	*Melopsittacus undulatus*	Germany	9	
IMT42073_Bird	*Melopsittacus undulatus*	Germany	9	**595**
IMT43363_Bird	*Melopsittacus undulatus*	Germany	9	
IMT40696_Dog	*Canis lupus familiaris*	Germany	16	596
3526_Vietnam_Pig	*Sus scrofa*	Vietnam	Non-typeable	**597**
3530_Vietnam_Pig	*Sus scrofa*	Vietnam	Non-typeable	
3505_Bird	*Gallus gallus*	Vietnam	9	**599**
3534_Bird	*Gallus gallus*	Vietnam	9	
IMT55097_Dog	*Canis lupus familiaris*	Germany	9	601
IMT55018_Cat	*Felis catus*	Germany	5	602
IMT50864_Bird	*Melopsittacus undulatus*	Germany	31	605
IMT49871_Bird	*Serinus canaria domestica*	Germany	9	606
IMT49673_Rat	*Rattus rattus*	Germany	Non-typeable	607
IMT49137_Bird	*Cyanistes caeruleus*	Germany	9	608
IMT47252_Cattle	*Bos taurus*	Germany	Non-typeable	609
IMT45178_Cattle	*Bos taurus*	Germany	23	610
IMT45074_Dog	*Canis lupus familiaris*	Germany	Non-typeable	611
IMT44590_Dog	*Canis lupus familiaris*	Germany	31	612
Fish	*Trichopodus pectoralis*	Thailand	6	613
Cat	*Felis catus*	Malaysia	8	614
3558_Bird	*Gallus gallus*	Vietnam	9	615
3545_Bird	*Gallus gallus*	Vietnam	9	622
3543_Bird	*Gallus gallus*	Vietnam	9	623
3542_Bird	*Gallus gallus*	Vietnam	Non-typeable	624
3538_Bird	*Gallus gallus*	Vietnam	9	625
3535_Bird	*Gallus gallus*	Vietnam	9	626
3524_Vietnam_Pig	*Sus scrofa*	Vietnam	4	632
3520_Bird	*Gallus gallus*	Vietnam	9	634
3518_Vietnam_Pig	*Sus scrofa*	Vietnam	31	635

*S. suis* isolates were serotyped in silico with the *S. suis* serotyping pipeline by Athey et al. [47]. Non-typeable isolates could not be typed with the pipeline. The fourth column shows the results for multi-locus sequence typing (MLST). All isolates except for the otter belong to novel MLST types. Lineages were determined by using PopPUNK and the genomes of the published *S. suis* database available online at https://www.bacpop.org/poppunk/ (downloaded: 04.08.23) and first described in Murray et al. [16, 48]. Lineages comprising more than one isolate are marked in bold. All isolates except for the otter isolate (pathogenic lineage 2) form new lineages distinct from the ones previously identified [16].

**Fig. 1:**
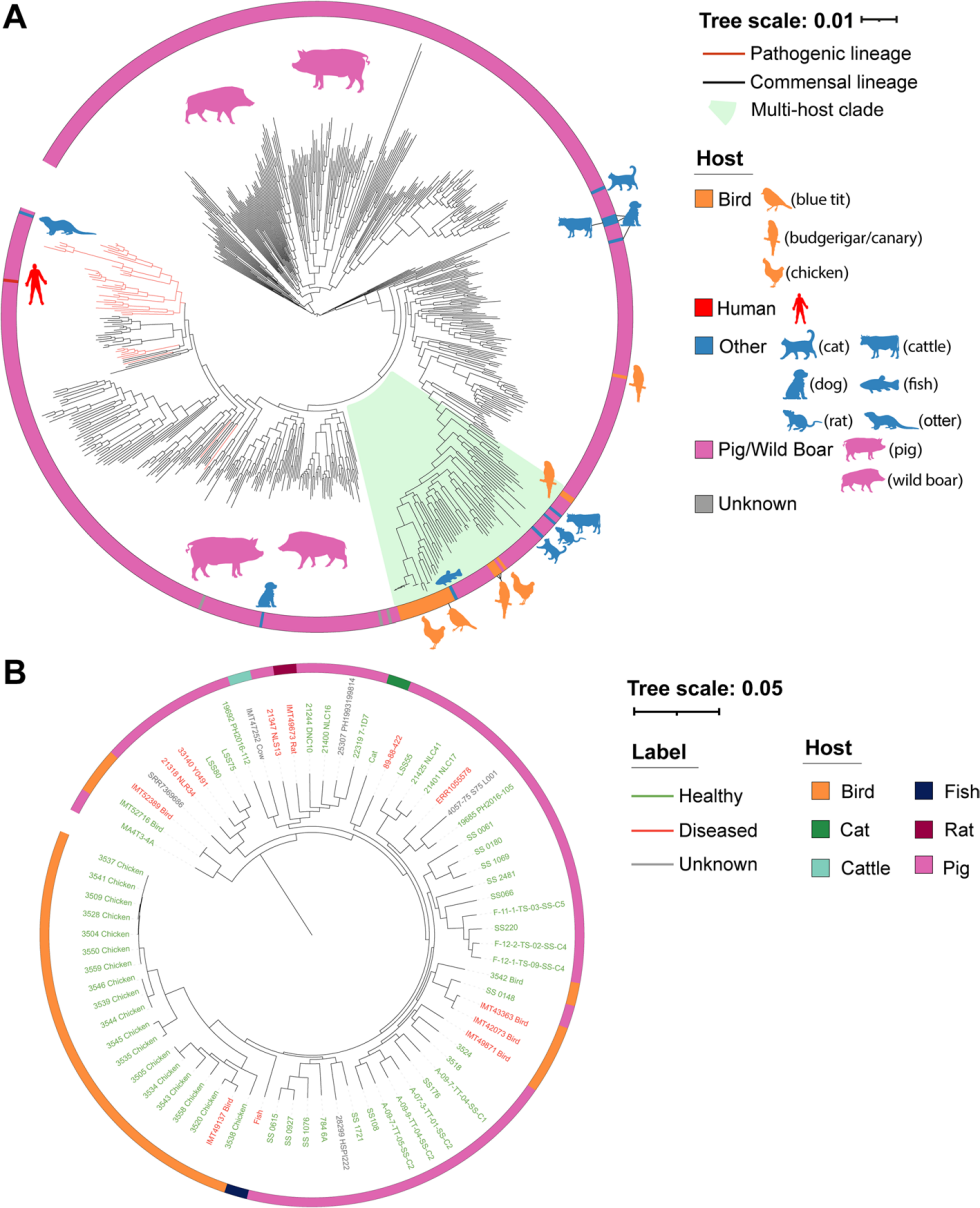
Isolates from bird and other species cluster with pig commensal isolates. Prokka v.1.13.3 was used to annotate the genomes [52]. Orthologous genes were identified, and alignments of the shared genes were created with Panaroo v.1.2.7 [53]*.* The maximum-likelihood phylogenetic tree was created using Fasttree, rooted at midpoint and visualised with ITOL [54, 55]. The tree scale represents the number of substitutions per site. **A** The tree includes one *S. suis* isolate of each lineage identified in Murray et al. [16], seven Vietnamese pig isolates and the *S. suis* isolates from birds and other species [23, 25, 26]. The host species is indicated in the outer ring. Silhouettes highlight the different host species. Branch colour indicates lineage belonging. **B** Multi-host clade (green coloured background in **A**) including the fish, the rat, one cat, one cattle and all bird isolates except for IMT50864. The outer ring shows the host association. Isolate names are coloured according to health status of the host, green for healthy and red for diseased. Metadata of the trees can be found in Tables S6 and S7 [52–54]

We then combined our data with a set of previously published genomes of *S. suis* from pigs, wild boars, and humans (Additional File 1: Table S5 [16]) to build a phylogenetic tree [16] (Fig. 1A). The otter isolate clustered with known pathogenic lineages which are shown as red branches in Fig. 1 and were identified by Murray et al. based on the presence of three pathogenic islands [16]. The other animal (non-pig) isolates significantly clustered together (permutation test; pairwise patristic distance = 0.06; *ρ* = 0.0089) within a group of commensal pig lineages. Clustering of animal isolates was largely driven by the clustering of bird isolates alone (permutation test; pairwise patristic distance = 0.04; *ρ* < 0.0001) (Additional File 2: Figure S3). Phylogenetic clustering is indicative of persistence and transmission among animal species [44].

To enhance visualisation of metadata and for further analysis, we pruned the tree at a node that contained a group of most of the bird and animal isolates. Except for one *S. suis* bird isolate (IMT50864) which was serotype 31, all other bird and chicken isolates as well as one cat, one cattle, the rat and fish isolate were part of this multi-host clade (Fig. 1B).

### Bird isolates resemble pig commensal isolates in their genome size and virulence

**Fig. 2:**
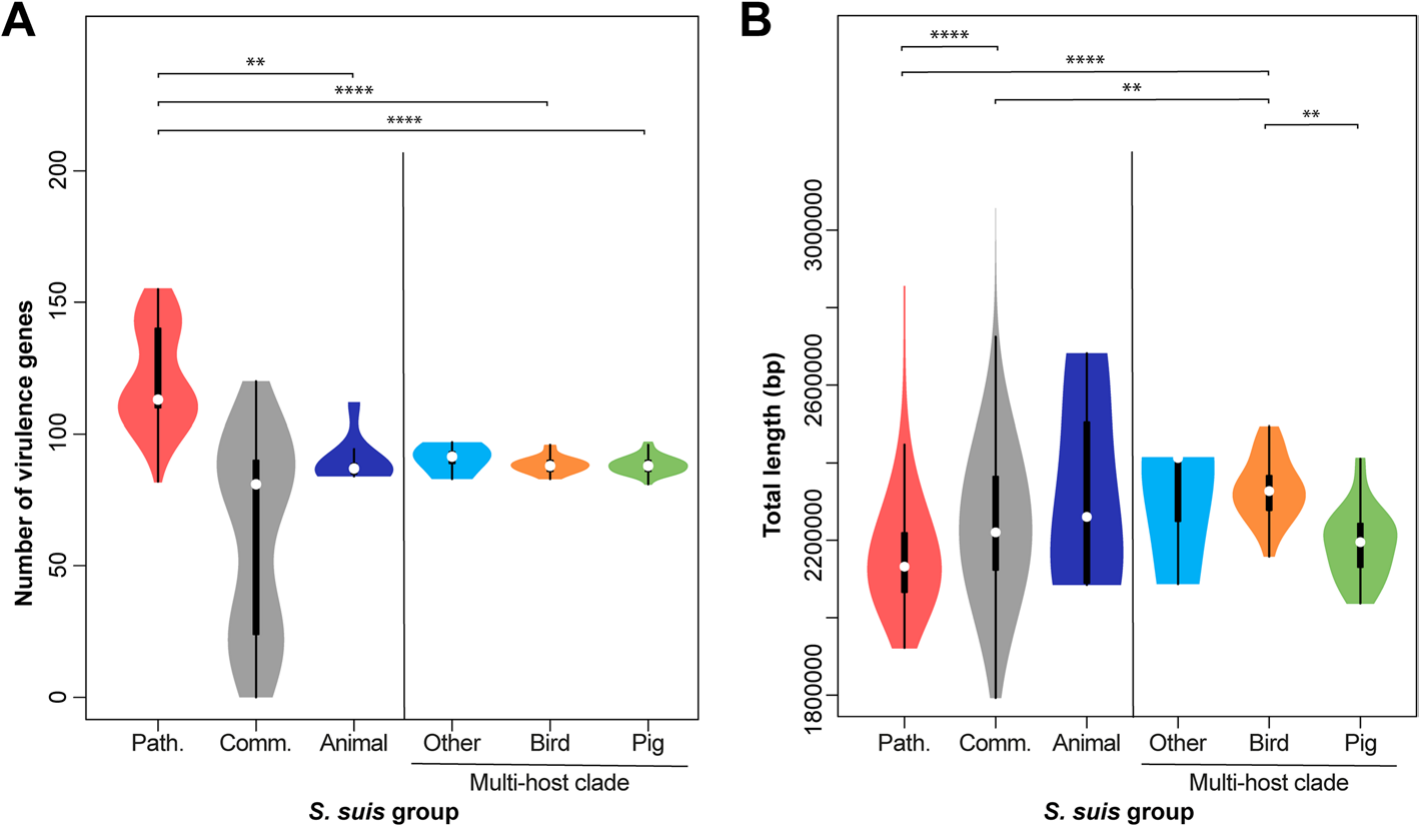
Bird isolates have less virulence genes, and a larger genome size compared to pathogenic *S*. *suis* lineages. **A** Virulence genes were analysed with ABRicate v.1.0.1 [56]. An *S.* *suis*-specific virulence gene list based on several publications was used as input [57]. **B** Total length of the genomes was analysed with QUAST [63]. The pathogenic group (red) comprises 1523, the commensal group (grey) 1554, the animal group (dark blue) 8, and the multi-host clade 72 isolates (other (light blue) = 4, bird (orange) = 25, pig (green) = 43). The white circle indicates the median of the data and the black box the interquartile range. The borders of the thin black line indicate the 1.5 interquartile range values. The cat isolate was excluded from the genome size analysis as its genome size of more than 4,000,000 bp indicated a mixture of isolates in this sequence. Data were analysed by Kruskal–Wallis test followed by Dunn’s multiple comparisons test. Significance is indicated by * *ρ* ≤ 0.05, ** *ρ* ≤ 0.01, *** *ρ* ≤ 0.001 and **** *ρ* ≤ 0.0001. Values and statistical results are shown in Tables S10-12

Our analysis suggests that poultry isolates are unlikely to have zoonotic potential given their phylogenetic clustering with pig commensal isolates and divergence from human zoonotic isolates. To investigate this in more detail, we investigated the presence of 170 *S. suis*-specific virulence genes (Additional File 1: Table S8 [56, 57]) in our isolate collection. *S. suis* isolates have a wide range of virulence genes that are associated with colonisation and survival within the host [18, 58, 59]. For this and the following analyses we defined six different groups of isolates: a pathogenic group containing pig, wild boar and human isolates from pathogenic lineages (1523 isolates) and a commensal group containing isolates from pigs, wild boar and environmental samples from commensal lineages (1554 isolates), both defined in Murray et al. based on the presence of three pathogenic islands [16] and an animal group containing all non-pig, non-human isolates outside the multi-host clade (eight isolates). The multi-host clade (Fig. 1B) (72 isolates) was divided into three different groups: the bird group (25 isolates), the other group containing all isolates from non-pig and non-bird hosts (four isolates), and a pig isolate group (43 isolates). The exact isolate composition of each group is shown in Additional File 1: Table S9 [16]. As shown before [60], isolates from commensal lineages possessed a lower number of virulence genes than those from the pathogenic group (Fig. 2A). The bird, other animal, and pigs from the multi-host clade had similar numbers of virulence genes to the commensal group, consistent with their phylogenetic placement. There were no virulence genes that were exclusive to the avian isolates (Additional File 1: Table S10 [16, 56] and S11 (statistics) [61, 62]).

Pathogenicity in *S. suis* also correlates negatively with genome size [57] and so we investigated variation in genome size using QUAST [63]. Consistent with previous studies [57, 60], the commensal group had a significantly larger genome size than the pathogenic group (Fig. 2B). On average, the other and bird group had the largest genome size but still fall within the range of the commensal group. Results are provided in Additional File 1: Table S12 [16, 63] and statistical tests in S11 [61, 62].

### Unique mobile genetic elements show host adaptation of S. suis to birds

**Fig. 3:**
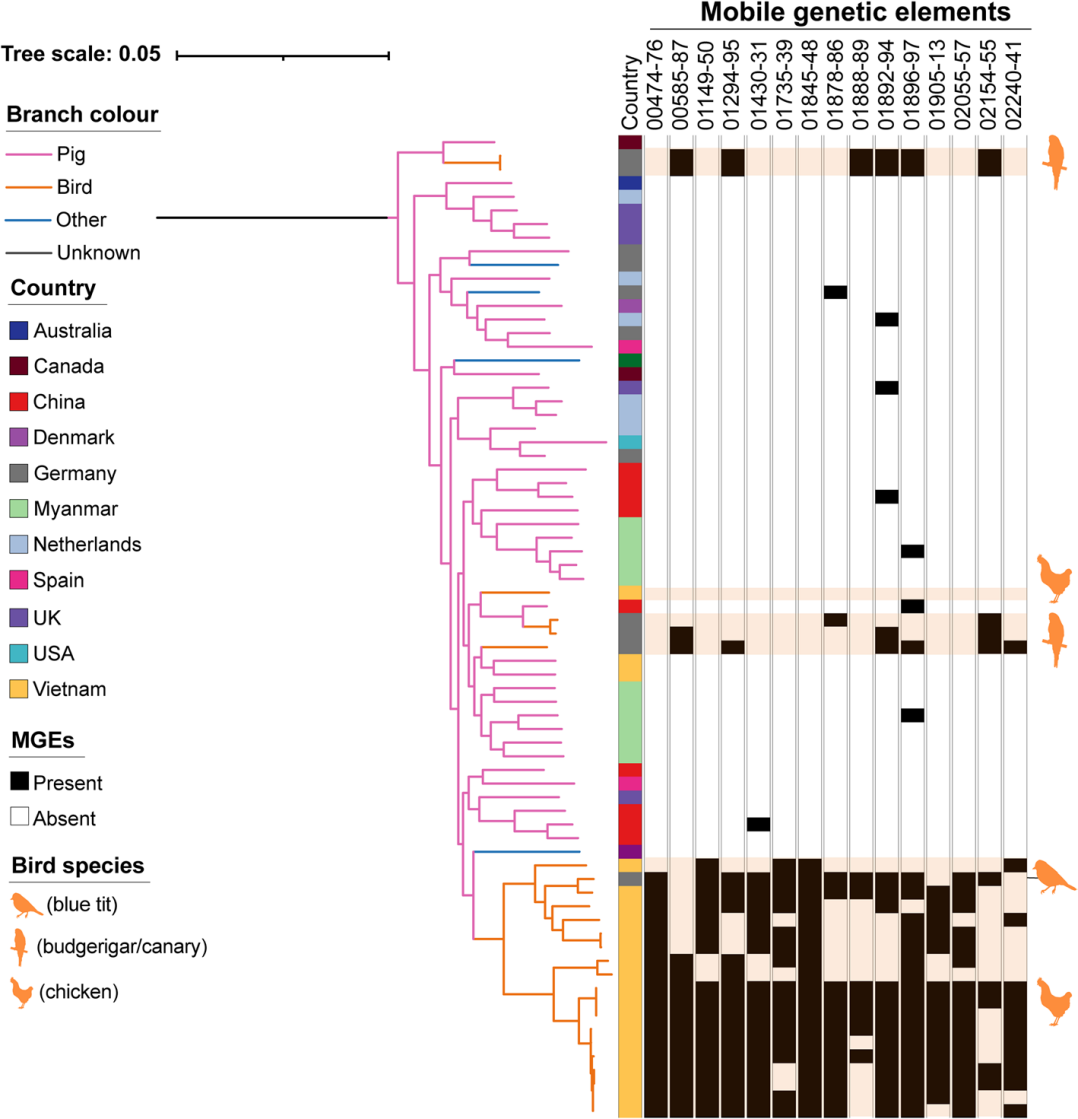
GWAS identifies 15 potential mobile genetic elements associated with *S. suis* bird isolates. GWAS analysis was performed with Scoary [64] and identified 15 mobile genetic elements (MGEs) associated with bird isolates. The presence of these MGEs is shown in the multi-host clade (pruned tree in Fig. 1B). The tree scale represents the number of substitutions per site. Tree branches are coloured according to host species. The first column shows the country of origin of the isolate and the remaining columns the presence (black) or absence (white) of the MGEs. The predicted functions of the MGEs are shown in Additional File 1: Table S16. Bird isolates are highlighted in light orange and with species silhouettes. The metadata of the tree is displayed in Additional File 1: Table S15

As our identified phylogenetic clustering of bird isolates is a hallmark of host adaptation [44], we performed a pan-genome-wide association study (panGWAS) with Scoary [64]. Gene content differences driven by mobile genetic elements (MGEs) have been shown to contribute to host adaptation and antibiotic resistance in different bacterial species, including streptococci [44, 65, 66]. We investigated whether isolates from birds differed in gene content from those from non-bird species, or if isolates from species other than pigs (including bird isolates) differed from pig isolates. Results are shown in Tables S13 and S14 [64]. In total we identified 61 overrepresented and four underrepresented genes in birds compared to other host species and 50 genes overrepresented in non-pig species compared to pigs, which completely overlapped with those identified in the comparison between bird and non-bird hosts (Bonferroni corrected *ρ*-value ≤ 0.05). Overrepresented genes in birds were classified as potential MGEs if two or more genes were neighbouring on the genome. This led us to identify 15 putative bird-associated MGEs that included 53/61 genes overrepresented in birds. Eight MGEs were found exclusively in birds and chickens. Of these one was found in chickens alone (MGE 01905–13), none in birds alone and seven in both (MGEs 00585–00587, 01149–01150, 01735–01739, 01845–01848, 01888–01889, 02055–02057, 02154–02155). The size of the MGEs ranged from 600 to 8226 bp. The smallest MGEs contained two genes, the biggest one nine genes. Only one of the 26 bird isolates (3542_Bird) did not contain any of these MGEs (Fig. 3). Although some of these MGEs were occasionally present in isolates from non-bird species, including pigs and cattle, on average the identified MGEs were present in 61% of the bird isolates in the multi-host clade, but only 1% of the non-bird isolates in this group. None of the identified MGEs were present in the Vietnamese pig isolates, suggesting that geographical structure is not the cause of this over-representation. In addition, the same MGEs were found over multiple countries. Some MGEs, e.g. MGEs 00585–087, 01294–01295 and 02154–55, have been acquired multiple times by *S. suis*. However, most of the MGEs were only present in one of the bird isolates from Germany, IMT49137, but most of the chicken isolates (Fig. 3, Additional File 1: Table S15 [52–54, 64]).

Given their repeated acquisition by lineages found in birds and distribution over different geographical locations (Germany and Vietnam), these MGEs may be providing *S. suis* with a fitness advantage in birds. Therefore, the possible functions of the MGEs were investigated with PROKKA annotation, BLAST and AlphaFold [52, 67, 68]. BLAST analysis [68] revealed that most closely related genes were found in publicly available *S. suis* or other streptococcal species such as *Streptococcus acidominimus* which has been isolated from slaughtered poultry [69]. One MGE (01905–01913) also comprised a gene with a close relative in the avian pathogen *Enterococcus cecorum* [70] (Additional File 1: Table S16 [52, 67, 68]). The genes carried by these MGEs had functions linked to metabolism, which can play a key role in streptococcal host adaptation, colonisation and the establishment of infection [71]. The MGEs 00474–00476, 01735–01739, 01845–01848 and 01905–01913 contained ABC transporter components involved in amino acid, vitamin B12 and iron transport and 01149–01150 contained another copy of the catabolite control protein A (CcpA), one of the main regulators of glucose metabolism and previously described as associated with the virulence of *S. suis* [72]. Due to their role in metabolism, ATP binding cassette (ABC) transporters can affect the fitness, as well as the virulence of bacteria [73]. The function of the genes on the remaining MGEs was mainly either unknown or linked to transcription and translation. Many genes were predicted integrases, transcriptional regulators or transposases. These factors mediate the uptake and distribution of MGEs to the genome of new hosts [74]. More detailed information on the functions of the MGEs is displayed in Additional File 1: Table S16. The genetic organisation of the MGEs is shown in Additional File 2: Figure S4 [53, 75, 76].

As surface proteins are most prone to directly interact with the host, we predicted potential signal peptides of the MGE-associated genes and their cleavage sites with SignalP [77]. Signal peptides are short recognition sequences that enable the transport of proteins across membranes [78]. SignalP predicted three potential signal peptides: 01430 (function unknown), 01894 (cell wall protein) and 01910 (ABC transporter substrate-binding protein) (Additional File 2: Figure S5 [77]). 01910 is the only gene that is part of an MGE exclusively present in the avian species. However, the substrate of this protein remains unknown.

### Bird isolates survive better in chicken blood than a commensal pig isolate

The phylogenetic clustering of isolates from birds and the presence of bird-associated MGEs suggest the adaptation of some *S. suis* lineages to birds. To investigate this phenotypically, we conducted ex vivo survival assays in chicken and pig blood using three genetically distinct bird isolates from different bird species (IMT49137 (blue tit), IMT49871 (canary), IMT50864 (budgerigar)) and three genetically distinct pig isolates as a control (pathogenic S10 [79], putatively pathogenic T15 [80] and commensal PH-105 [16]). This assay can also test pathogenesis of *S. suis* in birds as the ability to survive in blood is important for dissemination in the host [9]. The CFUs and the statistical results are summarised in Additional File 1: Table S17 [16].

**Fig. 4:**
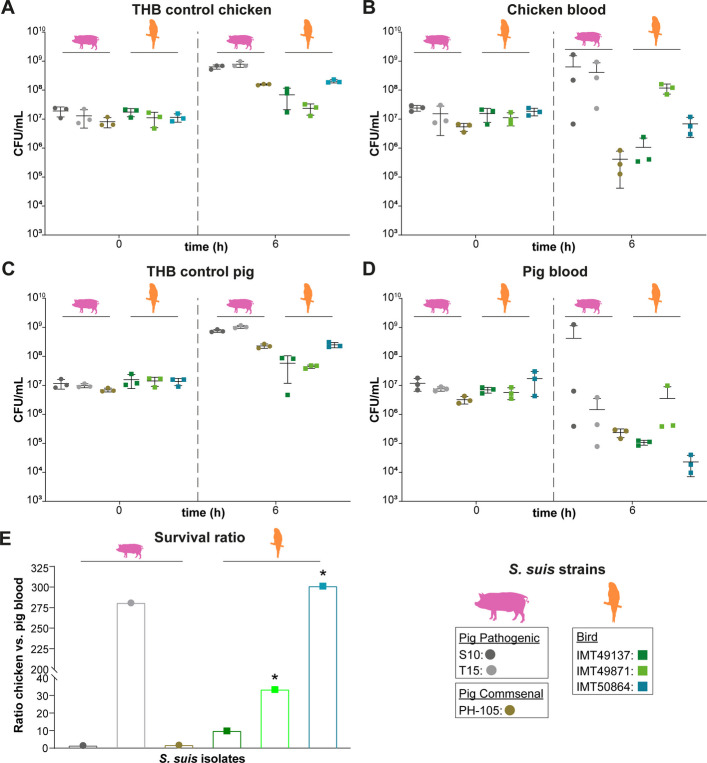
S. *suis* bird isolates tend to survive better in chicken than in porcine blood. Heparinised blood samples of chicken (upper panel) and pigs (middle panel), respectively, were inoculated with different *S. suis* bird isolates (IMT49137, IMT49871, IMT50864) or the porcine isolates S10, T15 and PH-105 as a control. S10 and T15 belong to a pathogenic lineage, whereas isolate PH-105 belongs to a commensal lineage. Pig isolates are depicted as circles, bird isolates as squares. Colony forming units (CFU) were determined by plating at time points t_0_ and t_6_. Data are presented as mean ± standard deviation of three independent experiments. **A** and **C** show control growth of *S. suis* isolates in THB medium for the respective experiments. **B** Survival in chicken blood. Blood of two individual animals was pooled for each experiment. **D** Survival in porcine blood. Blood of two animals was pooled for each experiment. **A**–**D, E** Survival ratio of the mean CFUs of different isolates in chicken vs. porcine blood after 6 h of incubation. Data were analysed by two-tailed paired samples *t*-test. Details of the statistical results can be found in Additional File 1: Table S17. Significance is indicated by an asterisk (*ρ* ≤ 0.05)

Pig isolates from the pathogenic lineage (S10 and T15) exhibited higher CFUs on average after 6 h relative to the commensal pig isolate (PH-105) in both pig and chicken blood. Pig isolates from the pathogenic lineage (S10 and T15) showed an increase in CFUs in chicken blood after 6 h compared to inoculation. S10 also showed an increase in the CFU in pig blood, whereas CFU values for T15 decreased. CFUs of the commensal pig isolate PH-105 decreased in both blood types (Fig. 4B, D). Both pathogenic isolates exhibited strong variation across different replicates indicating differences in the immune status of donor animals. While survival varied among bird isolates in chicken blood, IMT49871 showed higher CFUs after 6 h compared to inoculation. CFUs for IMT50864 remained stable but dropped for IMT49137. All three bird isolates showed higher CFUs after 6 h in chicken blood than the commensal pig isolate. In pig blood, two bird isolates (IMT49137 and IMT50864) had reduced survival compared to all pig isolates, whereas IMT49871 survived better than the commensal pig isolate. These findings suggest a degree of host-specific adaptation in the bird isolates for survival in chicken blood (Fig. 4B, D). Notably, two of the bird isolates demonstrated significantly better survival in chicken blood compared to pig blood after 6 h (Fig. 4E; statistical tests in Additional File 1: Table S17).

### Bird isolates are multi-drug resistant

Antimicrobial resistance (AMR) has been shown to be involved in host adaptation as well as interspecies jump events [81, 82]. We therefore investigated the presence of AMR genes and classes in the different groups with ABRicate [56]. Classes of AMR genes were determined based on the classes in the CARD database and the publication of Hadjirin et al. [83, 84] (Tables S18 and S19).

**Fig. 5:**
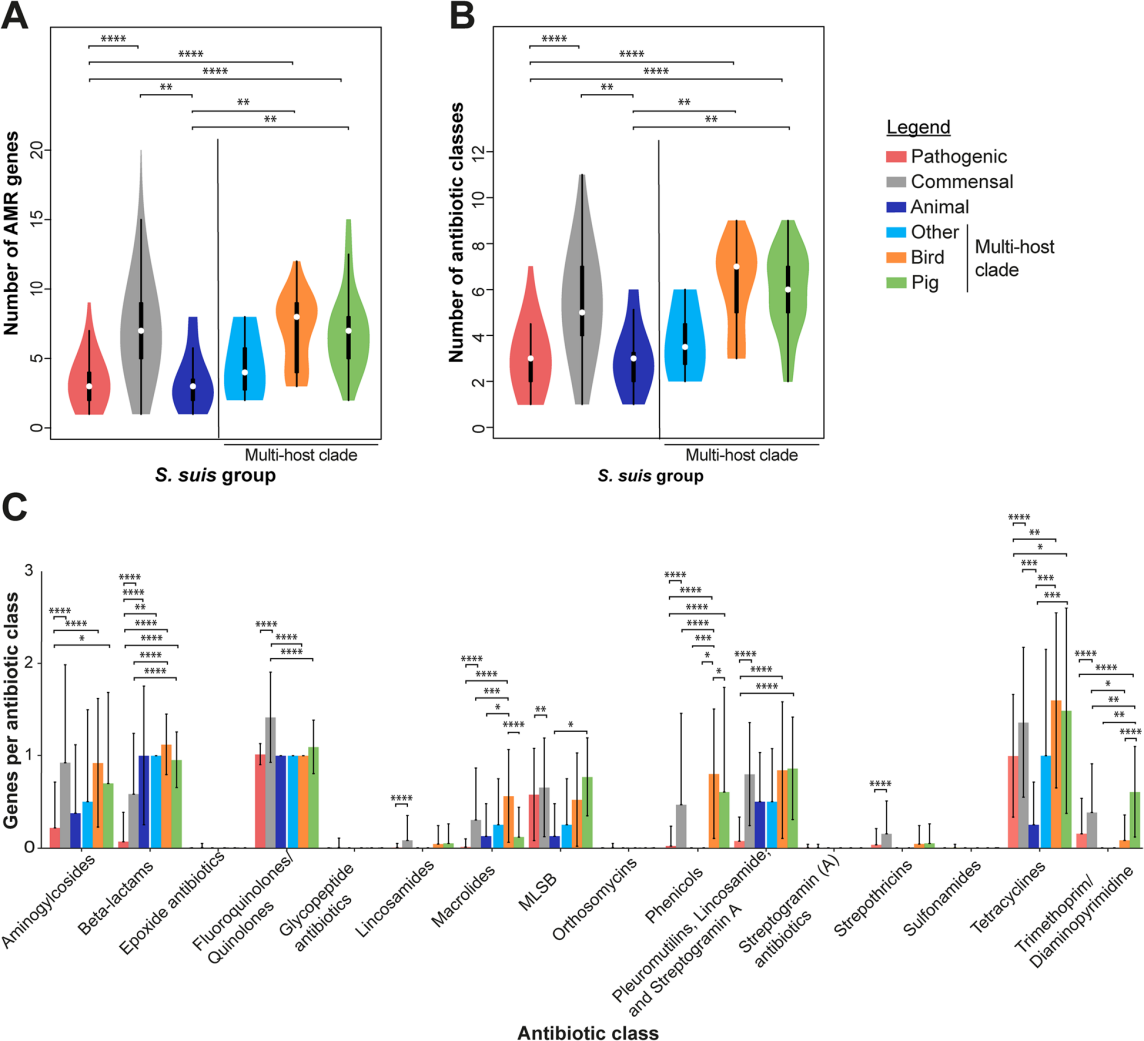
Bird isolates are all predicted to be multi-drug resistant and carry more antibiotic classes than any other group. Antimicrobial resistance (AMR) genes were analysed with ABRicate v.1.0.1 [56]. **A** shows the number of AMR genes per isolate. **B** shows the number of the different antibiotic classes per isolate. For a class to be counted as positive, at least one AMR gene in this class needed to be present per isolate. The white circles indicate the median of the data and the black box the interquartile range. The thin black line indicates the 1.5 interquartile range values. In **C**, average AMR gene numbers per class are shown across the five different groups. Data are shown as mean ± standard deviation. The pathogenic group (red) comprises 1523, the commensal group (grey) 1554, the animal group (dark blue) 8 and the multi-host clade 72 isolates (other (light blue) = 4, bird (orange) = 25, pig (green) = 43). Data were analysed by Kruskal–Wallis test followed by Dunn’s multiple comparisons test. Significance is indicated by * *ρ* ≤ 0.05, ** *ρ* ≤ 0.01, *** *ρ* ≤ 0.001 and **** *ρ* ≤ 0.0001. Details of the analyses and statistical results can be found in Tables S20 to S25 [16, 56, 61, 62, 83, 84]

Isolates from the pathogenic group and animal group possessed the fewest AMR genes (average of three per isolate), whereas the commensal and multi-host clade groups (except for other isolates) showed higher numbers (around seven per isolate) (Fig. 5A, Additional File 1: Tables S20-S22). The bird group possessed the highest diversity of AMR classes, closely followed by the commensal and pigs in the multi-host clade groups, while the pathogenic group had the least (Fig. 5B, Additional File 1: Table S23). A high proportion (> 68%) of isolates in the commensal, bird, and pig multi-host clade groups carried genes conferring resistance to five or more different AMR classes, compared to only 6.5% of pathogenic isolates and 18% of “other” isolates.

While resistance genes for fluoroquinolones/quinolones, macrolide/lincosamide/streptogramin B (MLSB), pleuromutilins, and tetracyclines were prevalent across all *S. suis* groups (drugs from these classes are commonly used in pig farming [5]), notable differences emerged for beta-lactam resistance, the standard treatment for *S. suis* infections [85]. The commensal, bird, and pig multi-host clade groups averaged at least one beta-lactam resistance gene (including genes positive for resistance relevant SNPs), whereas genes in this class were largely absent in pathogenic group (Fig. 5C, Tables S24 and S25). Analysis of alleles in pencillin-binding protein (*pbp*) genes, associated with beta-lactam resistance [83, 86], revealed that the highest number of alleles was present in *pbp2b*, particularly in the animal group and the multi-host clade (4/4) (Additional File 2: Figure S6 [83, 87], Additional File 1: Tables S26 and S27 [16, 61, 62]), suggesting potential mechanisms of reduced beta-lactam susceptibility in these groups. All bird isolates were predicted to be fluoroquinolone/quinolone resistant and bird isolates harboured the highest number of macrolide resistance genes on average. Both antibiotic classes are commonly used in the poultry industry in Vietnam where the chicken isolates originated from [25, 88].

### Independent host jump events from pigs to birds are associated with carriage of antimicrobial resistance genes

**Fig. 6:**
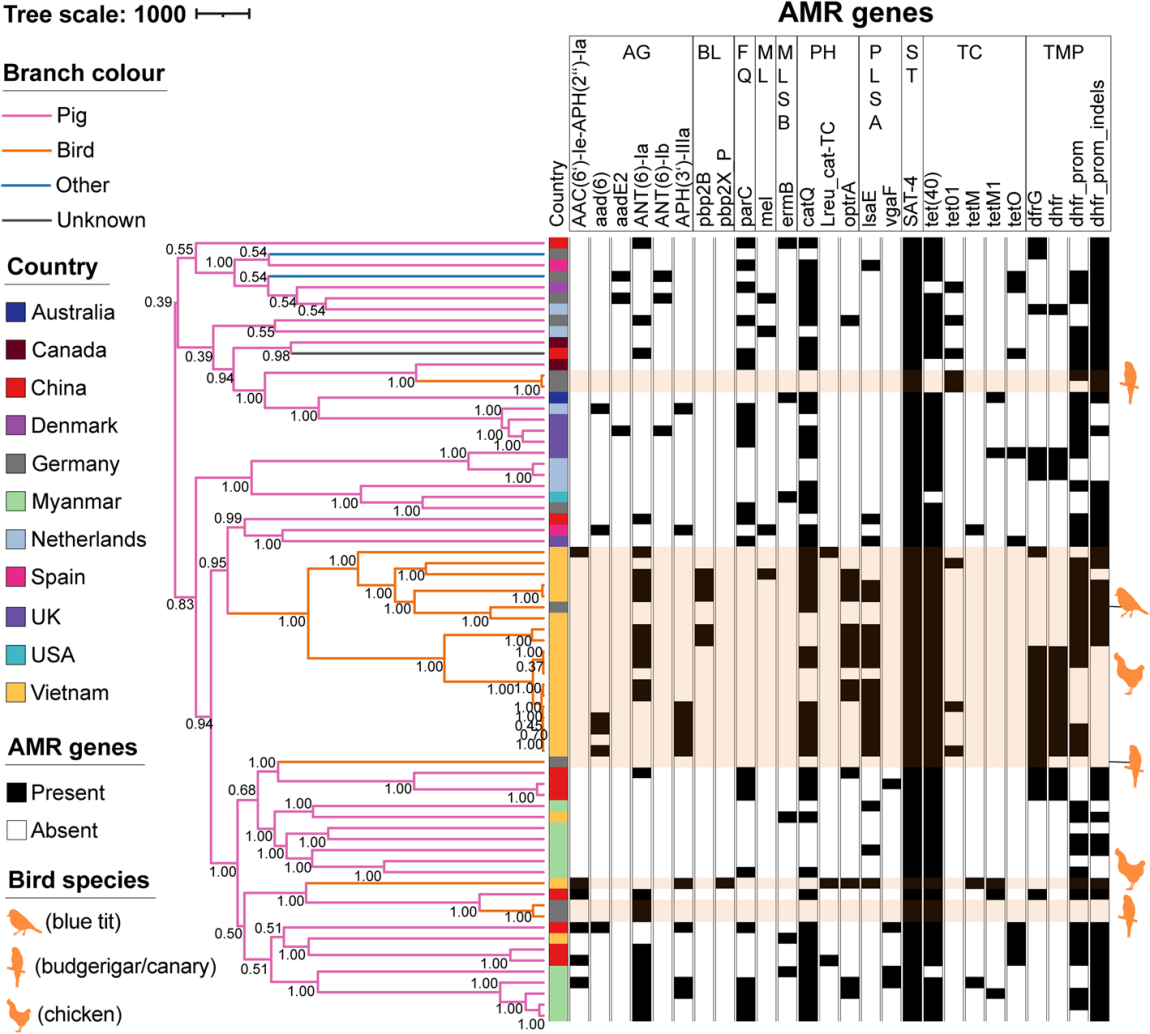
S. *suis* bird isolates show a dynamic antimicrobial resistance pattern. The tree was created with BEAST and visualised in ITOL [45, 54]. The tree scale represents the number of years. Tree branches are coloured according to host species: pig (pink), bird (orange), other (blue), unknown (grey). Node labels indicate posterior values for branch support. The first column shows the country of origin of the isolate and the remaining columns the presence (black) or absence (white) of antimicrobial resistance (AMR) genes associated with different AMR classes identified by ABRicate [56]. Bird isolates are highlighted in orange and with species silhouettes. The metadata of the tree is provided in Additional File 1: Table S28 [45, 54, 56, 87, 93]. AG = aminoglycosides; BL = beta-lactams; FL = fluoroquinolones; ML = macrolides; MLSB = macrolide, lincosamide, streptogramin B; PH = phenicols; PLSA = pleuromutilin, lincosamide, streptogramin A; ST = streptothricins; TC = tetracyclines; TMP = trimethoprim/diaminopyrimidine

We investigated the molecular epidemiology of the bird-associated *S. suis* group through phylogenetic analysis. The resulting phylogenetic reconstruction revealed five distinct instances of *S. suis* host jumps from pigs to birds (Fig. 6). One of these host transitions was a lot older than the other events, indicating an early establishment of *S. suis* in avian hosts followed by subsequent diversification and transmission within bird populations. Notably, a blue tit isolate from Germany clustered with chicken isolates from Vietnam, suggesting *S. suis* transmission from farmed poultry to wild birds across continents. Conversely, the presence of *S. suis* in pet birds appears to be a consequence of jumps from pigs. While most within-bird transmission events and some host jumps have occurred relatively recently (within the last 40 years), our analysis also identified older host-switching events (Additional File 2: Figure S7 [45, 54]). However, all our isolates were sampled very recently, which limits the strength of the dating analysis. Nevertheless, 95% HPD confidence intervals suggest the oldest node to be at least 6000 years old (Additional File 2: Figure S8 [89]).

As AMR can also be an important prerequisite for the adaptation to new host species [82, 90], we investigated the dynamics of AMR genes in the multi-host clade. Figures 5 and 6 show that most resistance genes, notably those conferring resistance to MLSB and tetracycline, were prevalent in bird isolates, mirroring observations in pig populations [83, 91]. The macrolide resistance gene *ermB* and tetracycline AMR genes are often co-transferred [92] and in around 40% of our bird isolates the AMR genes *ermB* and *tetO* were located on the same contig. This shared presence suggests that either antibiotic resistance genes in birds predate the host switch from pigs or that there is a similar usage pattern across the two host populations. Nevertheless, the predicted higher number of macrolide resistance genes (*mel* gene) and unique presence of some phenicol resistance genes, particularly evident in chicken isolates from Vietnam, coupled with the near absence of trimethoprim resistance, is suggestive of specific selective pressures within avian hosts.

## Discussion

Our study identified multiple host jump events of a multidrug-resistant *S. suis* group, originating in pigs, into various bird species, a cow, and a rat. The bird isolates are distinguished by a significantly higher frequency of AMR genes across a broader range of affected antimicrobial classes compared to other *S. suis* groups found in pigs or different animal hosts but still fall within the range of the commensal group. The observation that the frequencies of AMR genes in the avian isolates broadly mirror those in their porcine counterparts suggests that AMR gene acquisition might have predated these interspecies transmission events. Especially the presence in pet birds and the blue tit suggests acquisition of AMR genes before the host jump as these animals are not as often exposed to antibiotics as farm animals. However, as similar antibiotic classes are used in pig and poultry industry [94], gaining of new AMR genes in chicken is also plausible. The emergence and subsequent transmission of these *S. suis* lineages within avian populations have probably predominantly occurred within the last hundred years. Nonetheless, as bird populations are only poorly sampled these dates may be underestimated. The last century is notably characterised by the widespread intensification of antibiotic use in both human and veterinary medicine, which has run parallel to a corresponding global increase in AMR [2]. These trends lead to the hypothesis that AMR itself may have been a pivotal factor in promoting these jumps from pigs to birds and for ensuring long-term persistence especially in chickens.

The widespread application of similar classes of antimicrobials across different host populations can create a common selective pressure, thereby promoting interspecies transmissions and expanding the ecological range of resistant bacteria [81, 95]. Sharing of other AMR pathogens between livestock, humans and the environment have also been described, e.g., *Escherichia coli*, *Salmonella sp.* and *Clostridioides difficile* [96, 97]. While many interspecies transmission events will be transient “spillovers” without sustained host adaptation [26, 39, 42], they create opportunities for future host adaptation. Our study provides evidence that the carriage of AMR genes may allow bacteria to establish footholds in new host populations because we find evidence for the adaptation of *S. suis* to birds.

Firstly, we found phylogenetic clustering of bird isolates over geographical and ecological distances, including an instance of apparent transmission from a commercial poultry population in Vietnam to a wild bird in Germany, as well as the grouping of pet bird isolates from different German cities (separated by approximately 80 km). Generally, *S. suis* transmission within and between farms is linked to pig flow, but its presence has also been confirmed in flies [16, 98] which have been shown to carry *S. suis* for at least 5 days when experimentally infected [99]. The detection of this group in a wild bird is particularly noteworthy, as these animals may serve as an additional, potentially underappreciated, reservoir and transmission route. This could pose a possible infection risk to pig herds, especially outdoor-reared pigs, and humans, as it has been demonstrated for other pathogens, e.g. *Escherichia coli* or influenza viruses [100, 101]. However, sample numbers in our study were small, and more isolates from geographically diverse collections are needed to validate the results.

Second, most of the identified bird-associated MGEs carried genes with functions linked to metabolic activity. Metabolic adaptation is not only a hallmark for *S. suis* host adaptation, colonisation and establishment of infection [71], but also seen as a key adaptation mechanism in group B streptococci (GBS) [102]. Some of the genes in these MGEs had close relatives in the avian commensal *Streptococcus alactolyticus* and the pathobiont *Enterococcus cecorum* [70, 103] which might indicate adaptation to birds through the uptake of mobile elements from avian-adapted microbiota. Alternatively, this could be evidence of passive uptake of genetic material from these isolates by natural competence upon entering the avian host environment [104–106].

Finally, *S. suis* bird isolates showed some evidence of enhanced survival in chicken vs. porcine blood. It should be noted, however, that there was considerable inter-assay variation that might reflect a strong effect of the individual blood donor. This is in concordance with the fact that host-specific factors, e.g. age, breed or immune status of the pig strongly influence the onset and development of disease [11, 107]. Still, survival of the bird strains in chicken blood suggests the pathogen’s ability of haematogenous dissemination in the avian host. Indeed, *S. suis* has been isolated from inner organs, such as the kidney and spleen in different bird species [22].

While our study provides evidence of a link between AMR and host jumps, there may be other explanations for this resistant group being associated with host jumps. This group has a high genetic diversity evidenced by multiple genetic PopPUNK lineages and the formation of a new clonal complex [48]. Furthermore, this specific *S. suis* group possesses, on average, larger genome sizes when compared to other *S. suis* groups. An increased genome size is sometimes hypothesised to confer greater metabolic flexibility and facilitate better adaptation to new or fluctuating environments, as bacteria with highly restricted host ranges or specialised niches often exhibit smaller, more streamlined genomes [108]. *Salmonella* Typhimurium, a multi-host pathogen, comprises a larger genome than its close relative *Salmonella* Typhi, which is restricted to human infection [109]. However, the association between host range and genome size is not backed up by comparative analyses across diverse bacterial taxa [110].

The role of AMR in bacterial host jumps agrees with other studies on methicillin-resistant *Staphylococcus aureus*, showing AMR genes contributing to host jumps from cattle to humans and from human to pigs by facilitating persistence in the new host population [82, 111, 112]. While in two cases, the AMR genes were acquired in the new host species (pig and human) [82, 112], the AMR genes were maintained in the other jump from cattle to humans along with a human immune evasion gene cluster [112].

The main antibiotic classes administered in the pig and poultry industry are tetracyclines, polypeptides, macrolides and penicillins [94], which is reflected in the significantly higher resistance against these classes in the chicken isolates compared to the other groups (Additional File 1: Table S25). While the same AMR genes are present in pig as well as bird *S. suis* isolates, there are some genes more associated with bird isolates compared to the pig isolates in the multi-host clade in the classes beta-lactams (*pbp2b*), macrolides (*mel*) and phenicols (*catQ*), whereas trimethoprim AMR genes are nearly absent. These differences might be due to special selection pressures in the avian host. Amoxicillin is one of the most used antibiotics in poultry industry, and its application has been linked to co-selection of phenicol and beta-lactam AMR genes in chickens [113, 114].

It is important to acknowledge that genomic data does not always match phenotypic AMR data. A previous study found that there are few *S. suis* isolates with high phenotypic resistance that carry no corresponding AMR determinants, but many isolates show low phenotypic resistance despite the presence of a corresponding AMR determinant [83]. However, the high AMR of the chicken isolates in our study was confirmed via antimicrobial susceptibility testing in the study by Nhung et al. [25].

As the bird isolates formed part of the commensal group of *S. suis* and not of the zoonotic or highly virulent lineages, and consistent with their placement in this group carried less virulence genes than the pathogenic group, the immediate risk of zoonotic transmission from these avian-adapted isolates to humans is likely to be low. Commensal *S. suis* are often regarded as part of the natural flora of pigs and have not been associated with human disease [11, 16]. However, should this multidrug-resistant *S. suis* lineage acquire additional genetic traits that enhance its ability to efficiently transmit to and colonise humans, it could represent a future threat to public health, e.g. as a foodborne disease or via close contact to avian species. Until now clonal complex (CC) 1, which mainly consist of serotype 2 isolates, the leading cause of zoonotic infections in human, but recently, other CCs have been identified as causes for human disease [115, 116]. This suggests that the spectrum of *S. suis* isolates capable of causing zoonotic infections may be expanding, highlighting the importance of monitoring novel host adaptations.

The discovery of persistence of *S. suis* in a novel species is also critical because it can introduce genetic novelty to the pathogen [117]. The movement of *S. suis* between poultry and pigs could mean that antibiotic use in poultry affects resistance levels in pigs and contributes to the persistence of resistant lineages of *S. suis* in pigs. It has been suggested that *S. suis* acts as a source of antibiotic resistance genes for other bacterial pathogens, facilitating the dissemination of AMR genes among various streptococcal species [8, 118]. While we find no evidence of bird-to-pig transmission, it is limited by small sample size. Therefore, future research with a larger and more diverse dataset is needed to confirm *S. suis* jumps to and persistence in birds as well as to investigate whether transmission from birds to other species occurs.

## Conclusions

In conclusion, our study finds evidence of the repeated spillover into and occasional persistence of highly drug-resistant isolates of *S. suis* in chickens, pet birds and a blue tit. This could be promoted by a co-selection pressure for drug resistance or a disruption of the natural microbial ecosystems due to shared antimicrobial use in livestock and companion animals. Our results suggest that *S. suis* has adapted to birds, potentially promoting its onward transmission from poultry farms to wild birds and therefore the dissemination of this highly AMR bacterial species beyond populations subject to high levels of antibiotic exposure. The cross-species transmission of a highly drug-resistant bacteria is a significant public health concern and while there is no evidence that this lineage is currently either zoonotic or highly virulent, further evolution may lead to the emergence of a novel AMR pathogen in human, livestock or wild animal species. These findings highlight potential long-term risks of the co-use of antimicrobials in medicine and agriculture and how transfers of both bacteria and their AMR genes across species boundaries require monitoring outside of the context of AMR infections.

## Methods

### S. suis isolates and NCBI search

Between 2016 and 2024, in total seventeen *S. suis* isolates from species other than pigs, wild boar and humans (seven birds, four dog and four cattle, one cat and one rat) were isolated by the veterinary diagnostics department at the Freie Universität in Berlin. The isolates all originated from clinical samples sent in by veterinarians in the field. Detailed information on the isolates is summarised in Additional File 1: Table S2. Additional File 2: Figure S1 shows the sampling locations of the isolates in Germany. For the isolate IMT52389 only the post code of the pathology lab was available where the postmortem examination was carried out. The outline of the map of Germany showing the sample locations of the bird isolates was created in R v.4.2.3 [61] with the following packages: leaflet, rnaturalearth, rnaturalearthdata, sf and lwgeom [119–124]. The Germany map contains only isolates that were used for further analysis. Two cattle isolates were excluded (details below).

We combined theses isolates with a set of previously published *S. suis* genomes including 3070 high-quality genome assemblies. Detailed information on these isolates is found in the supplementary material from Murray et al. [16]. The sequencing data of these isolates are deposited in the European Nucleotide Archive (ENA) at EMBL-EBI under accession numbers PRJEB11219, PRJEB2670, PRJEB6250, PRJEB8392, PRJNA1009400, PRJNA1010137, PRJNA1012585, PRJNA428542, PRJNA476804, PRJNA628943, PRJNA763404 and PRJNA972671. Moreover, we included all publicly available *S. suis* genomes from species other than pigs, humans, wild boars, mice or the environment. The NCBI database was searched for *S. suis* sequences with the following search term: (((((("Streptococcus suis"[Organism] OR Streptococcus suis[All Fields]) NOT ("Homo sapiens"[Organism] OR Human[All Fields])) NOT ("Homo sapiens"[Organism] OR Homo sapiens[All Fields])) NOT ("Mus"[Organism] OR "Mus musculus"[Organism] OR Mouse[All Fields])) NOT ("Sus scrofa"[Organism] OR Pig[All Fields])) NOT ("Sus"[Organism] OR Sus[All Fields])) NOT Wastewater[All Fields] NOT culture[All Fields] NOT Sus scrofa[All Fields] NOT TSBS[All Fields] NOT lab[All Fields] NOT ("Sus scrofa"[Organism] OR swine[All Fields]) NOT ("unidentified"[Organism] OR unknown[All Fields]) NOT applicable[All Fields] NOT na[All Fields] NOT patient[All Fields] NOT collected[All Fields] NOT N/A[All Fields] AND "attribute host"[filter]. All downloaded sequences are summarised in Additional File 1: Table S3. We found isolates from a cat (Malaysia) [46], a fish [23], an otter (South Korea) [26], a lamb (Canada) [125], cattle (USA) [126] and 20 chicken isolates (Vietnam) [25]. We also included the Vietnamese pig isolates from the study from Nhung et al*.* as they were collected on the same or neighbouring farms as the chicken isolates [25].

### Whole genome sequencing and assembly

The isolates from Berlin were sequenced with Illumina whole genome sequencing technology. The libraries for WGS were prepared using the Nextera XT DNA Library Preparation Kit (Illumina, Inc., San Diego, USA) according to the manufacturer’s recommendations. The 2 × 300 bp paired-end sequencing in 40-fold multiplexes was performed on the Illumina MiSeq platform (Illumina, Inc., San Diego, USA) with MiSeq Reagent Kit v3 (600-cycle). The Illumina reads of the Berlin, chicken and the cat isolates were adapter-trimmed with Trimmomatic to remove adaptors and trim poor-quality ends. The following parameter settings were used: ILLUMINACLIP:TruSeq3-PE.fa:2:30:10:2:True LEADING:3 TRAILING:3 MINLEN:36 [127]. Afterwards, the short reads were de novo assembled using SPAdes v.3.12.0 with default parameter settings [128]. All isolates sequenced in Berlin were between 80 × and 220 × coverage. QUAST v.5.2.0 was used to evaluate these assemblies [63]. Moreover, the short reads were mapped back to the de novo assemblies with Bowtie2 v.2.3.4.1 to examine polymorphism, an indicator for mixed cultures [129]. For the fish and the otter isolate, only the assembled genomes were available online. After quality control [63] (Additional File 2: Figure S9 [63]) and additional checks with FastANI [130], we removed the cattle, lamb, one of the chicken and multiple Vietnamese pig isolates because they were phylogenetically divergent to the central *S. suis* population leaving 40 non-pig animal isolates (Additional File 2: Figure S10 [16, 23, 25, 26, 52–55]). PopPUNK v2.6.0 was used to cluster the genomes by investigating the variation in the core and accessory genes [48]. For clustering, we used the published *S. suis* database available online at https://www.bacpop.org/poppunk/ (downloaded: 04.08.23) and first described in Murray et al. [16]. Genome size of *S. suis* isolates was determined with QUAST v.5.2.0 [63]. Allelic profiles of the strains were analysed with PubMLST and clonal complexes with the PubMLST *S. suis* dataset and PHYLOViZ Online which is based on the goeBURST algorithm [49–51]. Isolates with no known sequence type were assigned one with consecutive numbers. Clonal complexes were defined as sequence types that only differed in one of the seven alleles, so-called single locus variants. Non-grouping isolates were defined as singletons [15, 17] (Additional File 2: Figure S2, Additional File 1: Table S4 [49–51]).

### In silico serotyping

The Berlin and other isolates for which short-read data was available were serotyped in silico using the Athey et al. pipeline which also detects the virulence genes *mrp*, *epf* and *sly* [47]. For the fish and the otter isolates (for which short-reads were not available), Illumina sequence reads (HiSeq 2500) were simulated with the sequencing read simulator ART v 2.6.0 [131] before analysing these isolates with the serotyping pipeline [47].

### Pangenome construction and phylogenetic analysis

To produce a phylogenetic tree of all isolates and examine accessory gene content, we randomly chose one isolate of each of 589 genetically distinct lineages identified in Murray et al. [16] and built a pangenome. First we used Prokka v.1.13.3 to annotate the genomes with default settings [52]. Orthologous genes were identified, and alignments of the shared genes were created with Panaroo v.1.2.7 using the recommended parameter settings [53]. A phylogenetic tree was created with Fasttree v 2.1.10 using the Jukes_cantor + CAT model and the -gamma option [55]. The tree was visualised with ITOL v.6.7.3 [54]. Colour strip metadata was produced with itolparser v0.1.1 [132]. Two isolates from Berlin (IMT53434 and IMT54075 from cattle) as well as 32 of the 39 Vietnamese pig isolates formed a clear outgroup in the phylogenetic tree (Additional File 2: Figure S10 [16, 23, 25, 26, 52–55]). Therefore, these isolates were excluded from further analysis and the Panaroo pipeline was run again after their removal with following analyses using this dataset.

Phylogenetic cluster analysis of the isolates (either birds or other species) was performed by using randomisation tests. For this, we applied a permutation-based approach [133]. The patristic distances between all samples were calculated from the phylogenetic tree shown in Fig. 1A and performed with the ape package in R [87]. In a next step, the median distance of either all bird isolates, bird and other species or only other species was calculated. To simulate the null distribution of the test statistic, the host association of all samples included in the phylogenetic tree was randomly permuted 10,000 times. Finally, the median of the average patristic distance was determined. The *p*-value was calculated by determining the proportion of permutation means that were smaller than the observed mean for the bird isolates.

### Pan-genome-wide association study

The pan-genome-wide association study was performed with Scoary v 1.6.16 [64]. Gene presence/absence was investigated for its association with the host origin in other species or birds. Genes overrepresented in bird isolates (Bonferroni *p*-value < 0.05) were screened for mobile genetic elements and their hypothetical function. Results are depicted in Tables S13 and S14. Identified genes were checked for their position on the genome with the gene presence/absence file obtained from the Panaroo output [53] and the SPAdes results file [128]. The isolate 3539_Bird was used as reference as it contained the most detected genes. Adjacent genes on the genome were summarised to identify potential mobile genetic elements (MGEs), and BLASTn was used as a second control to confirm presence/absence in the isolates. Functions of the MGEs were investigated using the PROKKA annotation file, online BLAST analysis and AlphaFold [52, 67, 68]. In addition, SignalP 6.0 was used to predict potential signal peptides and their cleavage sites [77].

### Survival in chicken and porcine blood

For the survival experiments in blood, the following *S. suis* pig isolates were used: pathogenic *S. suis* serotype 2 isolate 10 (S10) [79], *S. suis* T15 [80], which has been experimentally identified to be non-pathogenic but belongs to a pathogenic lineage according to Murray et al. [16] both kindly provided by H. Smith (Lelystad, the Netherlands), and a commensal pig isolate from the multi-host clade, isolate 19685_PH2016-105 (PH-105), isolated from the tonsil of a healthy pig in Germany [16]. Moreover, we chose three *S. suis* bird isolates (IMT49137, IMT49871, IMT50864) from different bird species. Growth experiments in chicken and porcine blood were performed as previously described [134]. In brief, bacterial isolates were cultured overnight at 37 °C on Columbia agar supplemented with 7% (v/v) sheep blood (Thermo Fisher Scientific, Waltham, MA, USA) under aerobic conditions. For each experiment, a starting culture was inoculated with five to six single colonies in Todd-Hewitt Broth medium (THB; Bacto™, Becton Dickinson, Franklin Lakes, NJ, USA) and incubated at 37 °C overnight. The next day, fresh THB medium was inoculated with the overnight cultures and bacterial cultures were adjusted to an optical density at 600 nm (OD_600_) of 0.02 in fresh THB medium and incubated to an OD_600_ of 0.2. Then, 10 mL of bacterial suspension was pelleted (4696 × *g*, 10 min, 4 °C), washed once with PBS buffer (Sigma-Aldrich, St. Louis, MO, USA) and adjusted to a concentration of approximately 2 × 10^8^ colony-forming units (CFU) per mL in PBS. Heparinised blood samples were inoculated with the corresponding isolate at a concentration of 1 × 10^7^ CFU/mL. Samples were incubated under gentle rotation for 6 h at 37 °C. CFU was determined by plating before incubation (time point zero) and after 6 h of incubation time. Experiments were performed with six biological replicates. For each experiment blood of two different animals was pooled. Chicken blood samples were taken from ten to 14-months year old SPF White Leghorn. Porcine blood samples were taken from 11- to 15-week-old hybrid pigs (German Landrace x German Large White) that were experimentally infected with *Mycoplasma hyopneumoniae*. Animals showed either no or mild disease symptoms like coughing. Prevalence of *M. hyopneumoniae* is very high in the pig population with antibodies in nearly 90% of the herds and a seroprevalence of up to 100% [135]. Therefore, *M. hyopneumoniae* free pigs are very rare. THB medium was used as a control.

### Virulence and antimicrobial resistance genes

To detect the number of virulence and antimicrobial resistance (AMR) genes, we tested different pipelines on a known sample including ARIBA, SRST2, AMRFinder plus and ABRicate [56, 136–138]. Since ABRicate gave the most reliable results, we continued the analysis with this software v.1.0.1 [56]. ABRicate performs the analysis on assembled genomes/contig files. The pipeline was run with default parameter settings. ABRicate reports all genes with a minimum coverage and identity of 80% as positive. For the virulence gene analysis, we created a separate database. Analysed virulence genes and their sequences are listed in Additional File 1: Table S8. This list is based on several publications, contains 170 virulence genes and was previously described in Murray et al. [57]. To find potential AMR genes, we used the Comprehensive Antibiotic Resistance Database (CARD) v.3.2.6 downloaded on 10.05.23 [84]. As not all *S. suis* AMR genes are listed in this database, we also included a supplementary database in our analysis based on the AMR determinants detected in Hadjirin et al. [83]. The additionally included AMR genes and their sequences are listed in Additional File 1: Table S18 [83, 84]. For both analyses, we included the whole *S. suis* population comprising 3070 isolates and published in Murray et al. [16]. The dataset was divided into pathogenic and commensal isolates based on the publication [16]. The classification of the groups is listed in Additional File 1: Table S9. To detect the AMR relevant allele variants, we generated reference-mapped assemblies with Bowtie2 v.2.3.4.1 using the isolate *S. suis* P1/7 (Genbank ID: AM946016.1) as a reference [129, 139]. For the other *S. suis* reference isolates, we needed to simulate Illumina sequence reads (HiSeq 2500) with the sequencing read simulator ART v 2.6.0 [131] as only the full genome sequences were available online. The combined alignment was loaded into R where the isolates were screened for the presence of the relevant alleles using the package ape [61, 87]. The used R script is available at https://github.com/M-Dresen/Strep-suis-Allele-mapping. For the *dhfr* promoter indels, positive hits from the publication by Hadjirin et al. [83] as well as deletions in this region were included as positive hits in this study. Mutations in the *dhfr* promoter were detected after reference mapping with R [61]. For genes with multiple allele variants, a gene was counted as positive when at least one allele was present in the respective gene. Antibiotic classes are based on Hadjirin et al. [83] and were complemented with positive hits for classes in the CARD database. The classes with the included genes are listed in Additional File 1: Table S19 [83, 84]. The number of AMR classes was determined using R [61].

### Molecular epidemiology

Most of the bird isolates and some isolates from other species clustered in one part of the *S. suis* phylogenetic tree, so we used this multi-host clade for molecular epidemiology analysis. The cat and fish isolate had to be removed as there was a discrepancy between their clock rates and phylogenetic informative sites. Furthermore, to investigate the molecular epidemiology of this lineage, we produced a reference-mapped assembly of the multi-host clade shown in Fig. 1B with Bowtie2 v.2.3.4.1 using the isolate D12 as a reference (Genbank: NC_017621.1) [129, 140]. D12 was the closest reference genome to the multi-host clade. We identified regions of recombination using Gubbins v3.3.1 with an adjusted filter percentage of 29% (filter out taxa with more than this percentage of gaps, default 25%) to include all isolates [93] and kept only non-recombined sites that had less than 10% gaps. Gaps were removed with the ape package in R [87]. After removal of recombination and gaps, 13% of the genome remained. To investigate temporal signal, we analysed the resulting tree with the software TempEst v1.5.3, which investigates the relation between genetic divergence over time and sample isolation dates [89] (R squared = 1.8641E-2), and using a published R script [141]. D12 was excluded for the TempEst analysis as its isolation year is unknown [140]. As we did not detect any temporal signal in both analyses (Additional File 2: Figure S11 [16, 87, 89, 93, 129]), we fixed the clock rate for the following BEAST analysis. We performed BEAST analysis (v1.10.4) with the following settings: uncorrelated relaxed clock model with defined clock rate (1E-6 substitutions/site/year (average clock rate of six different lineages in Murray et al. [16]), HKY substitution model with gamma site heterogeneity model, and a UPGMA starting tree. We created a separate partition of the trait host species and performed ancestral state as well as state change count reconstruction on this partition. Default priors, auto-optimised operators and a chain length of minimum 300,000,000 were used [45]. Out of three runs, two converged on the same optimum and were combined to one tree after removing burn-in. All ESS parameters exceeded 180. The results were robust to different model approaches. The country of origin, identified AMR genes as well as classes were plotted on the Beast tree using ITOL v.7 [54]. Colour strip metadata was produced with itolparser v0.1.1 [132]. Height_95%_HPD confidence intervals of the resulting tree are shown in Additional File 2: Figure S8.

### Statistical analysis and graphs

Statistical analysis was performed with base R v4.2.3 and the package dplyr [61, 62]. Data were analysed by Kruskal–Wallis test and if needed followed by Dunn’s multiple comparisons test. Data analysis was done separately for each antibiotic class in Fig. 5C. Blood survival ratios in Fig. 4E were analysed by two-tailed paired samples *t*-test after log10 transformation of the CFUs. Sample size is given in figure legends. Violin plots were produced with the R package vioplot and histograms with base R [61, 142]. Figure 4 was created with GraphPad PRISM Software 8.01 for Windows (GraphPad Software, San Diego, CA, USA). 

## Supplementary Information

Additional file 1: Additional File 1 contains all the supplementary tables mentioned in the manuscript. These tables contain information on the used dataset (Tables S1-5), metadata of the phylogenetic trees (Tables S6, S7, S15 and S28), detailed background information on the performed analyses (Tables S8, S9, S18 and S19), as well as the detailed results and statistics of these analyses (Tables S10-S17, S20-27).

Additional file 2: Additional File 2 contains all the supplementary figures mentioned in the manuscript. Figure S1 shows the sampling locations of the German bird isolates. Figure S2 show the clonal complexes of the animal and bird *S. suis* isolates. Figure S3-S8 show additional analyses results (phylogenetic clustering, mobile genetic element organisation, signal peptide predictions, antimicrobial resistance, and additional BEAST trees). Figure S9 and S10 show the quality control results and Figure S11 the absence of temporal signal in the pruned tree group.

## Data Availability

All data generated or analysed during this study are included in this published article. The sequencing data and genome assemblies for this study have been deposited in the European Nucleotide Archive (ENA) at EMBL-EBI under accession number PRJEB85928 (http://identifiers.org/ena.embl:PRJEB85928). The allele mapping R script is available on Github: M-Dresen, Strep-suis-Allele-mapping (https://github.com/M-Dresen/Strep-suis-Allele-mapping).

## Abbreviations

AMR: Antimicrobial resistance
ArcA: Arginine deiminase
ABC transporter: ATP binding cassette transporter
CcpA: Catabolite control protein A
CC: Clonal complex
CFU: Colony forming units
CARD: Comprehensive Antibiotic Resistance Database
EF, epf: Extracellular factor
GBS: Group B streptococci
Hyl: Hyaluronidase
MLSB: Macrolide, lincosamide, streptogramin B
MGEs: Mobile genetic elements
MLST: Multi-locus sequence typing
MRP: Muraminidase-released protein
*M. hyopneumoniae*: *Mycoplasma hyopneumoniae*
panGWAS: Pan-genome-wide association study
PBP: Penicillin-binding protein
Bay046: Protective antigen
SLY: Suilysin
*S. suis*: *Streptococcus suis*
THB: Todd-Hewitt Broth
USA: United States of America
