# Author Correction: *Streptococcus suis* avian expansion suggests shared antibiotic use drives host jumps

**DOI:** 10.1186/s12915-026-02692-7

**Published:** 2026-07-28

**Authors:** Muriel Dresen, Gemma G. R. Murray, Peter Valentin‑Weigand, Marcus Fulde, Lucy A. Weinert

**Affiliations:** 1 Department of Veterinary Medicine, Institute of Microbiology and Epizootics, Freie Universitat Berlin, Berlin, Germany; 2 Department of Veterinary Medicine, University of Cambridge, Cambridge, UK; 3 Department of Genetics, Evolution and Environment, University College London, London, UK; 4 Institute for Microbiology, University of Veterinary Medicine Hannover, Hannover, Germany

**Author Correction: BMC Biol 23, 358 (2025)**

**https://doi.org/10.1186/s12915-025–02477-4**

In this article, citations to one of the Supplementary Tables and citations to some of the references were incorrect.

The sentence “On average, the other and bird group had the largest genome size but still fall within the range of the commensal group. Results are provided in Additional file 1: Table S12 [16, 63] and statistical tests in **S10** [**16, 56**].” was corrected to “On average, the other and bird group had the largest genome size but still fall within the range of the commensal group. Results are provided in Additional file 1: Table S12 [16, 63] and statistical tests in **S11** [**61, 62**].”

The sentence “One MGE (01905–01913) also comprised a gene with a close relative in the avian pathogen *Enterococcus cecorum* (**Additional File 1: Table S16** [**52, 67, 68, 70**]).” was corrected to “One MGE (01905–01913) also comprised a gene with a close relative in the avian pathogen *Enterococcus cecorum* [**70**] (**Additional File 1: Table S16** [**52, 67, 68**]).”

The sentence “D12 was excluded for the TempEst analysis as its isolation year is **unknown**. As we did not detect any temporal signal in both analyses (**Additional File 2: Figure S11** [**16, 87, 89, 93, 129, 140**]), we fixed the clock rate for the following BEAST analysis.” was corrected to “D12 was excluded for the TempEst analysis as its isolation year is **unknown [140]**. As we did not detect any temporal signal in both analyses (**Additional File 2: Figure S11** [**16, 87, 89, 93, 129**]), we fixed the clock rate for the following BEAST analysis.”

The sentence “Additional file 1: Additional file 1 contains all the supplementary tables mentioned in the manuscript. These tables contain information on the used dataset (Tables S1-5), metadata of the phylogenetic trees (Tables S6, S7, S15 and S28), detailed background information on the performed analyses (Tables S8-**S10**, S18 and S19), as well as the detailed results and statistics of these analyses (Tables S10-S17, S20-27).” was corrected to “Additional file 1: Additional file 1 contains all the supplementary tables mentioned in the manuscript. These tables contain information on the used dataset (Tables S1-5), metadata of the phylogenetic trees (Tables S6, S7, S15 and S28), detailed background information on the performed analyses (Tables S8, **S9**, S18 and S19), as well as the detailed results and statistics of these analyses (Tables S10-S17, S20-27).”

In addition, some errors in the reference numbers and two table heading errors within Additional file 1 and Additional file 2 were corrected.

The original article has been corrected.

