# Supplementary material for: Streptococcus suis avian expansion suggests shared antibiotic use drives host jumps
Source: BMC Biol. 2025 Dec 2;23:358. doi: 10.1186/s12915-025-02477-4 (PMC12690903; doi:10.1186/s12915-025-02477-4)
Supplement: Supplementary file 2 — Additional file 2: Additional File 2 contains all the supplementary figures mentioned in the manuscript. Figure S1 shows the sampling locations of the German bird isolates. Figure S2 show the clonal complexes of the animal and bird S. suis isolates. Figure S3-S8 show additional analyses results (phylogenetic clustering, mobile genetic element organisation, signal peptide predictions, antimicrobial resistance, and additional BEAST trees). Figure S9 and S10 show the quality control results and Figure S11 the absence of temporal signal in the pruned tree group. [file 12915_2025_2477_MOESM2_ESM.pdf]

Streptococcus suis avian expansion suggests shared antibiotic use drives host jumps

Muriel Dresen[1,2]*, Gemma G. R. Murray[3], Peter Valentin-Weigand[4], Marcus Fulde[1,4], Lucy A. Weinert[2]

[1] Department of Veterinary Medicine, Institute of Microbiology and Epizootics, Freie Universität Berlin, Berlin, Germany

[2] Department of Veterinary Medicine, University of Cambridge, Cambridge, UK

[3] Department of Genetics, Evolution and Environment, University College London, London, UK

[4] Institute for Microbiology, University of Veterinary Medicine Hannover, Hannover, Germany

*Correspondence: muriel.dresen@fu-berlin.de

Additional file 2

1. Figure S1. Sampling locations of *Streptococcus suis* in birds in Germany.
2. Figure S2. Fifteen bird and one dog isolate form new clonal complex
3. Figure S3. Isolates from birds and other species cluster phylogenetically.
4. Figure S4: Genetic organisation of the 15 mobile genetic elements (MGEs).
5. Figure S5. SignalP predicts three (lipoprotein) signal peptides.
6. Figure S6: Number of *pbp* gene alleles per isolate.
7. Figure S7. BEAST analysis identifies five independent host jump events to birds.
8. Figure S8. Height_95%_HPD confidence intervals of BEAST tree.
9. Figure S9. Quality control analysis with Quast.
10. Figure S10. Thirty-two of the Vietnamese pig isolates and two cattle isolates form an outgroup in the phylogenetic tree.
11. Figure S11. No temporal signal detected with TempEst.

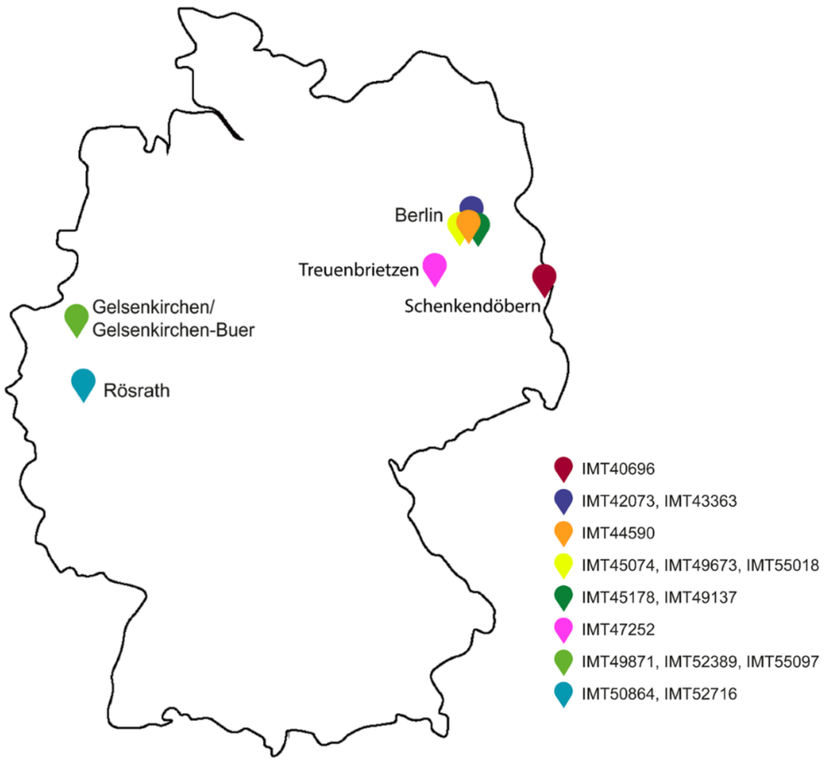

Figure S1. Sampling locations of *Streptococcus suis* isolates from Berlin in Germany. From 2016 until 2024 15 *S. suis* isolates from species other than pigs were sampled by field veterinarians who send the samples for analysis to the diagnostics department of the Department of Veterinary Medicine, Institute of Microbiology and Epizootics at the Freie Universität Berlin. These samples included seven bird, four dog, two cattle, one rat and one cat isolate. Sampling locations in Germany are marked with pins. The isolates originated either from the Eastern or Western part of Germany. For the isolate IMT52389 only the post code of the pathology lab not the sampling location was available where the postmortem examination was carried out.

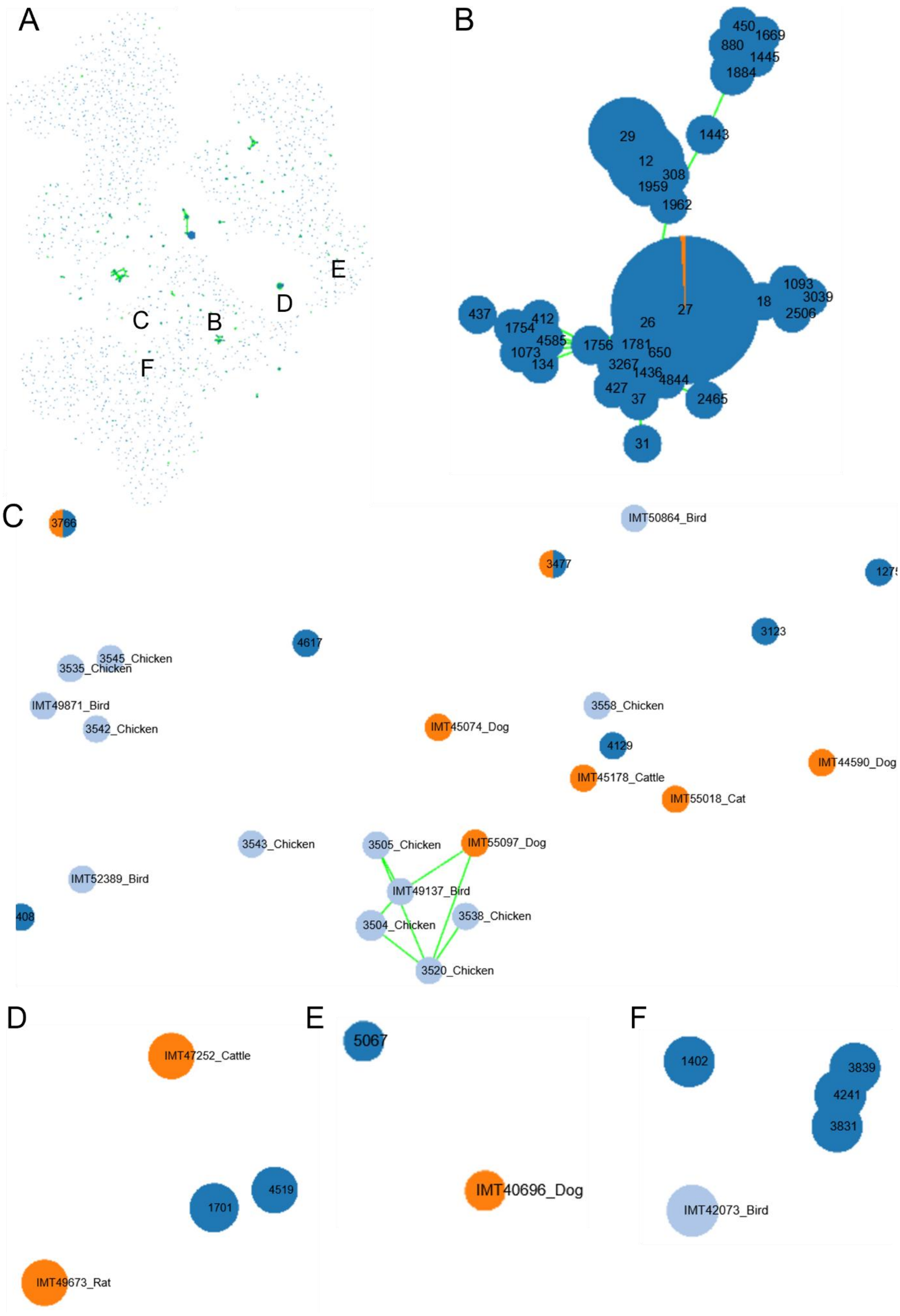

Figure S2. Fifteen bird and one dog isolate form new clonal complex. An *S. suis* dataset of more than 5000 isolates was downloaded from NCBI and combined with the chicken isolates and the isolates from Berlin. Allelic profiles were analysed with PubMLST and clonal complexes with PHYLOViZ Online

which is based on the goeBURST algorithm (49-51). (**A**) shows the overview of the grouping of all isolates and (**B**-**F**) the regions where own isolates were located. PubMLST isolates are depicted in dark blue, avian isolates in light blue and animal isolates in orange. CCs are linked by green lines. Small letters in (**A**) indicate locations on the map. (**C**) shows the new clonal complex, CC 4014, formed by 15 avian isolates and one dog isolate. Only one isolate per sequence type is represented on the map. Details of the sequence types and the new clonal complex are shown in Table S4.

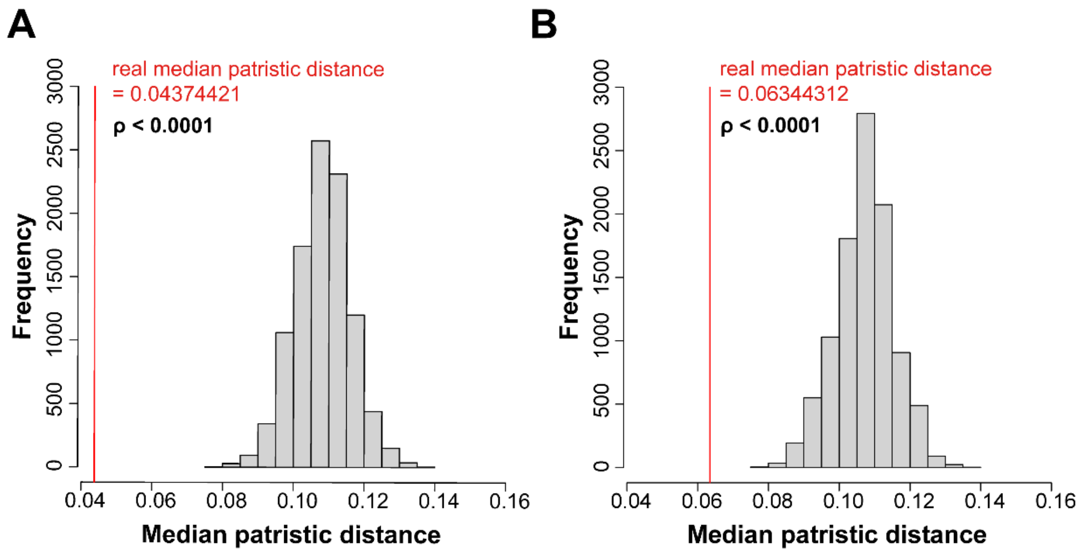

Figure S3. Isolates from birds and other species cluster phylogenetically. Histograms showing clustering of bird isolates (**A**) and isolates from birds and other species (**B**) across the phylogeny. The evolutionary (patristic) distance between isolates was calculated from the tree in Figure 1A. The average distance between all corresponding isolates was calculated. The null distribution was obtained by 10,000 randomisations of permuting host association of strains on the phylogenetic tree and by calculating the median of the average patristic distance. The true median is shown as a red line. The *ρ*-value represents the proportion of the permuted data sets with a median larger than the real median obtained with the tested isolates.

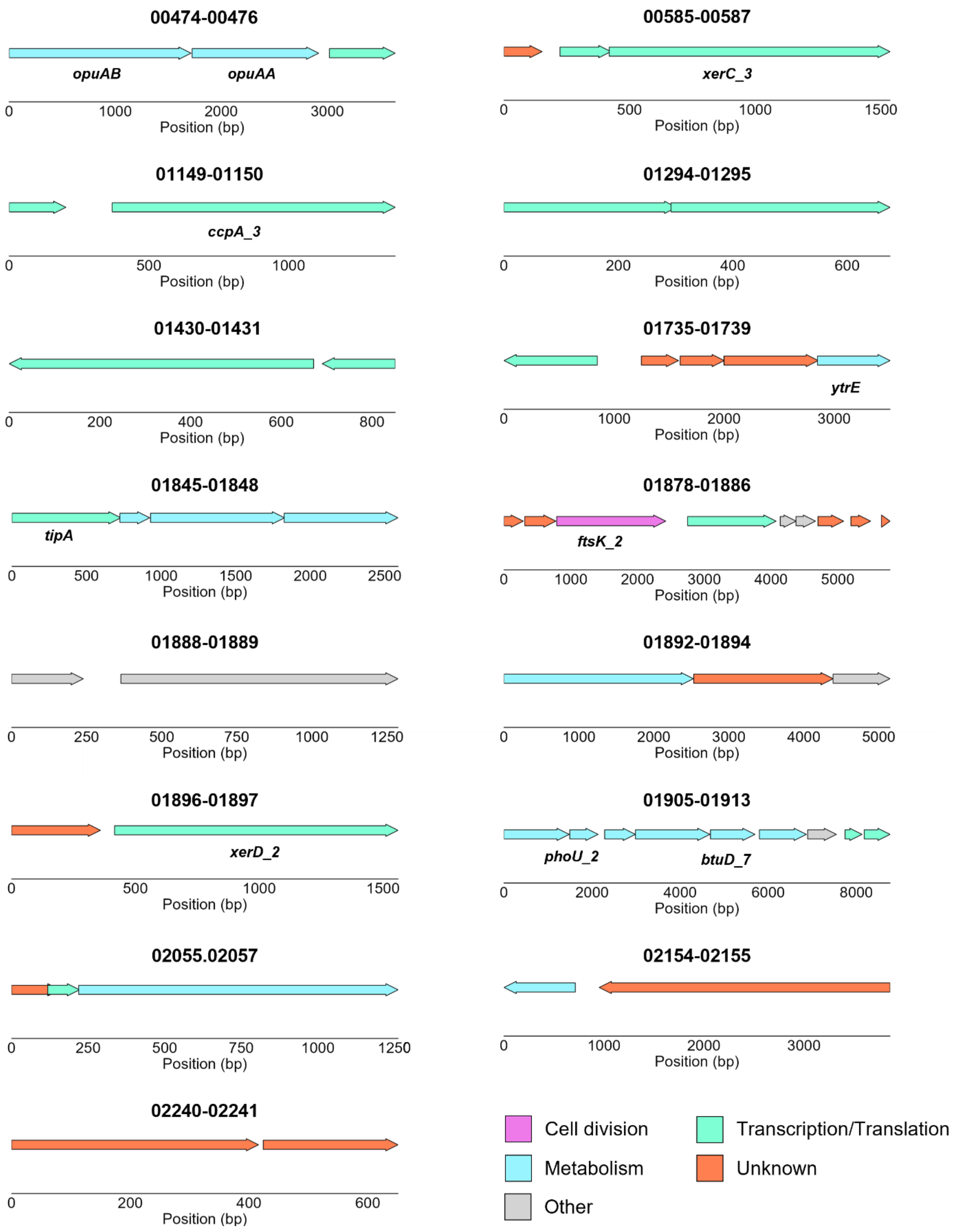

Figure S4. Genetic organisation of the 15 mobile genetic elements (MGEs). Plots show genomic organisation of the different MGEs. Rightwards arrows indicate genes on forward strand, leftwards arrows indicate genes on complement strand. The colour of the arrows indicates their predicted function. Gene names identified by Panaroo are indicated (53). Maps were generated in R with the packages ggplot2 and gggenes (75, 76).

A

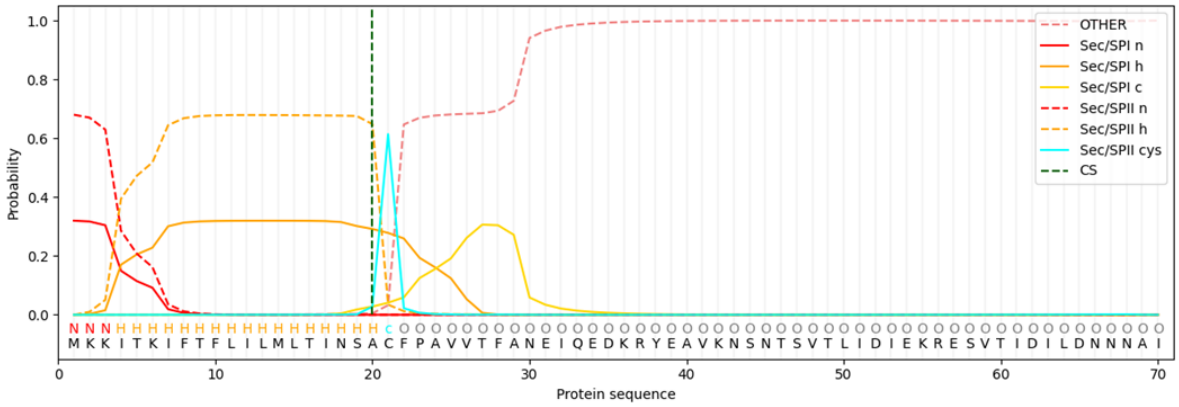

# SignalP-6.0	Organism: Other	Timestamp: 20241017131321						
# ID	Prediction	OTHER	SP(Sec/SPI)	LIPO(Sec/SPII)	TAT(Tat/SPI)	TATLIPO(Tat/SPII)	PILIN(Sec/SPIII)	CS Position
CHAPJJKG_01430	LIPO	0.000566	0.313475	0.685356	0.000261	0.000178	0.000144	CS pos: 20-21. Pr: 0.6483

B

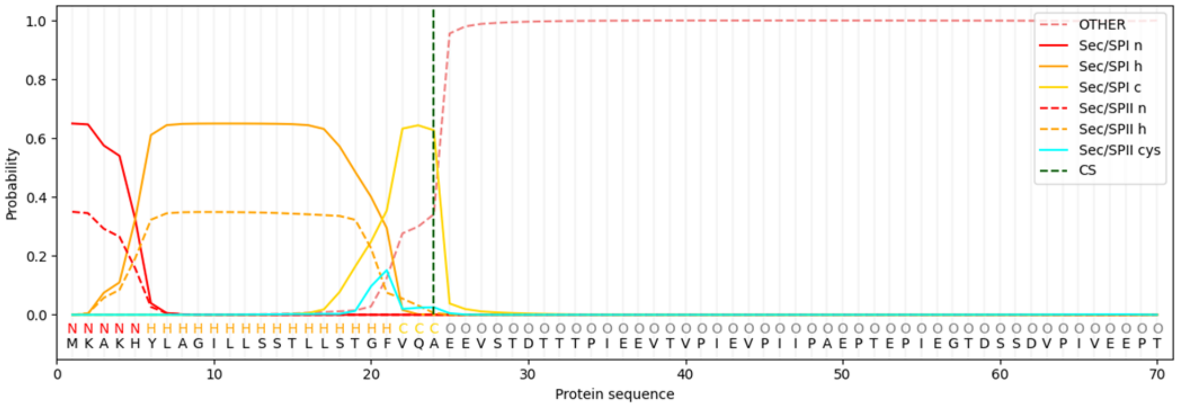

# SignalP-6.0	Organism: Other	Timestamp: 20241017140805						
# ID	Prediction	OTHER	SP(Sec/SPI)	LIPO(Sec/SPII)	TAT(Tat/SPI)	TATLIPO(Tat/SPII)	PILIN(Sec/SPIII)	CS Position
CHAPJJKG_01894	SP	0.001058	0.640021	0.357975	0.000395	0.000305	0.000217	CS pos: 24-25. Pr: 0.6270

C

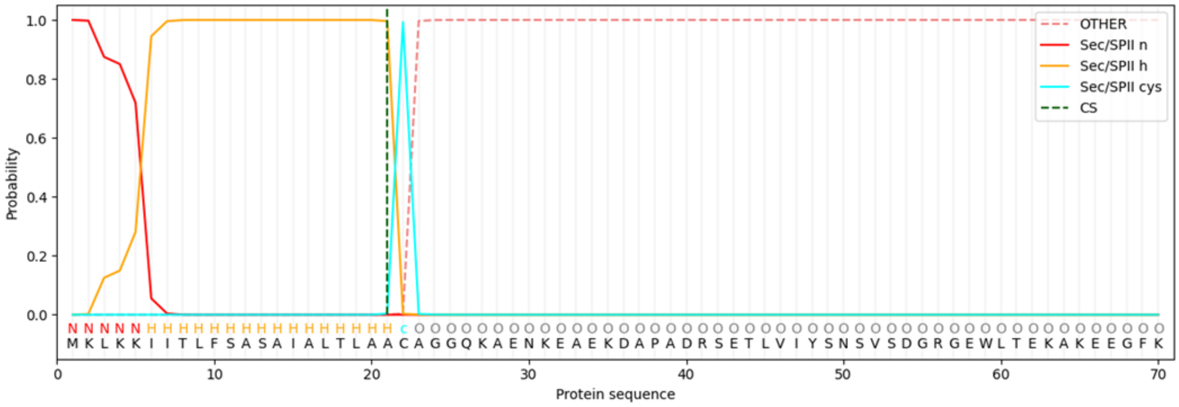

# SignalP-6.0	Organism: Other	Timestamp: 20241017143802						
# ID	Prediction	OTHER	SP(Sec/SPI)	LIPO(Sec/SPII)	TAT(Tat/SPI)	TATLIPO(Tat/SPII)	PILIN(Sec/SPIII)	CS Position
CHAPJJKG_01910	LIPO	0.000000	0.000000	1.000000	0.000000	0.000000	0.000000	CS pos: 21-22. Pr: 0.9961

Figure S5. SignalP predicts three (lipoprotein) signal peptides. The single proteins of the identified mobile genetic elements (MGEs) were analysed with SignalP 6.0 (77). (**A**) shows the results for 01430, a hypothetical protein, (**B**) the results for 01894, a cell wall anchor protein and (**C**) the results for 01910, an ABC transporter substrate binding protein. The predicted cleavage site is indicated by the green dashed line. 01430 and 01910 were predicted to be lipoprotein signal peptides (LIPO) and 01894 a Sec signal peptide (SP).

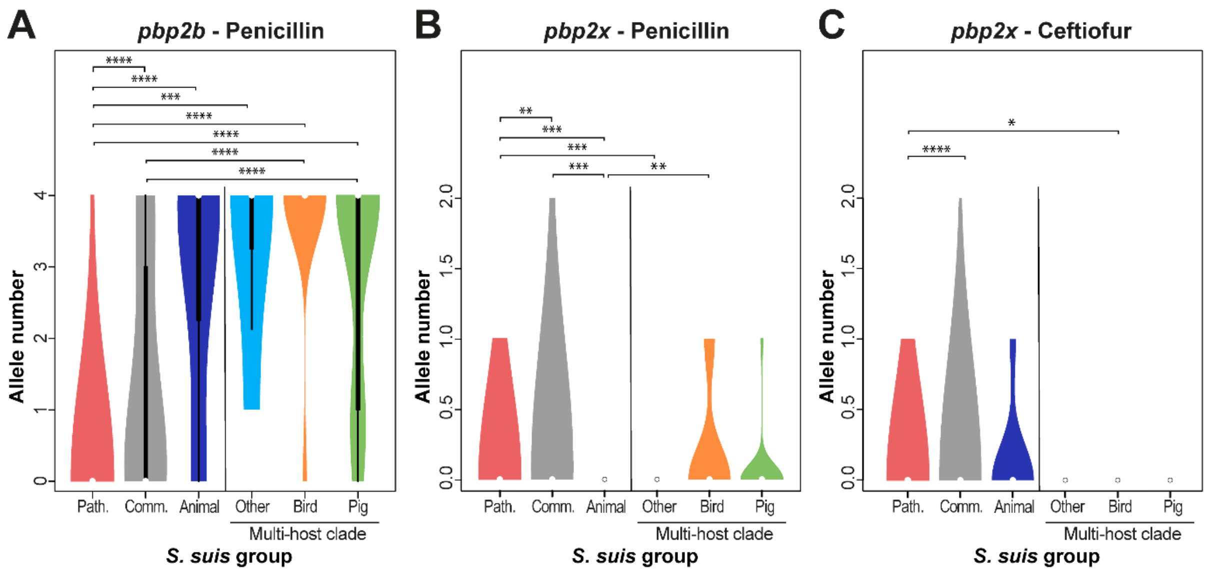

Figure S6. Number of *pbp* gene alleles per strain. Two different *pbp* genes were detected in the investigated *S. suis* isolates, *pbp2b* and *pbp2x*. Whereas allele changes in *pbp2b* lead to penicillin resistance, changes in *pbp2x* can either also lead to penicillin or ceftiofur resistance (83). Allele mutations were detected by generating reference-mapped assemblies with Bowtie2 using the strain *S. suis* P1/7 as a reference (129, 139). Presence or absence of allele variants was investigated with the ape package in R (87). In (**A**) the number of alleles per group in the *pbp2b* gene is shown. (**B**) and (**C**) show the allele number relevant for penicillin or ceftiofur resistance in the *pbp2x* gene. The pathogenic group (red) comprises 1523, the commensal group (grey) 1554, the animal group (purple) 8, the other group (blue) 4, the bird group (orange) 25, and the pruned tree group (green) 43 isolates. Data were analysed by Kruskal-Wallis test followed by Dunn's multiple comparisons test. Significance is indicated by * $\rho \leq 0.05$, ** $\rho \leq 0.01$, *** $\rho \leq 0.001$ and **** $\rho \leq 0.0001$. Details of the analysis and statistical results can be found in Tables S26 and S27.

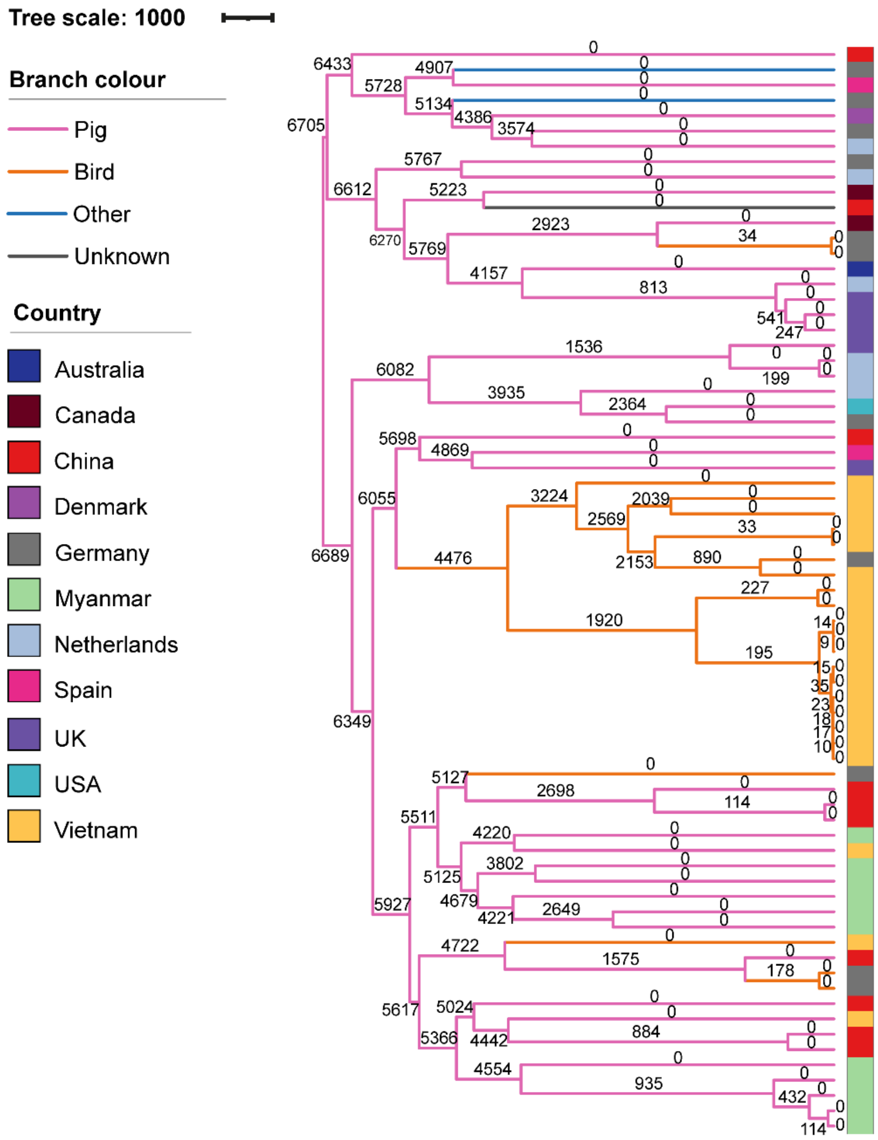

Figure S7. BEAST analysis identifies five independent host jump events to birds. The tree was created with BEAST and visualised in ITOL (45,54). Tree branches are coloured according to host species: pig (pink), bird (orange), other (blue), unknown (grey). The tree scale represents the number of years. Node labels indicate heights. The metadata of the tree is listed in Table S28.

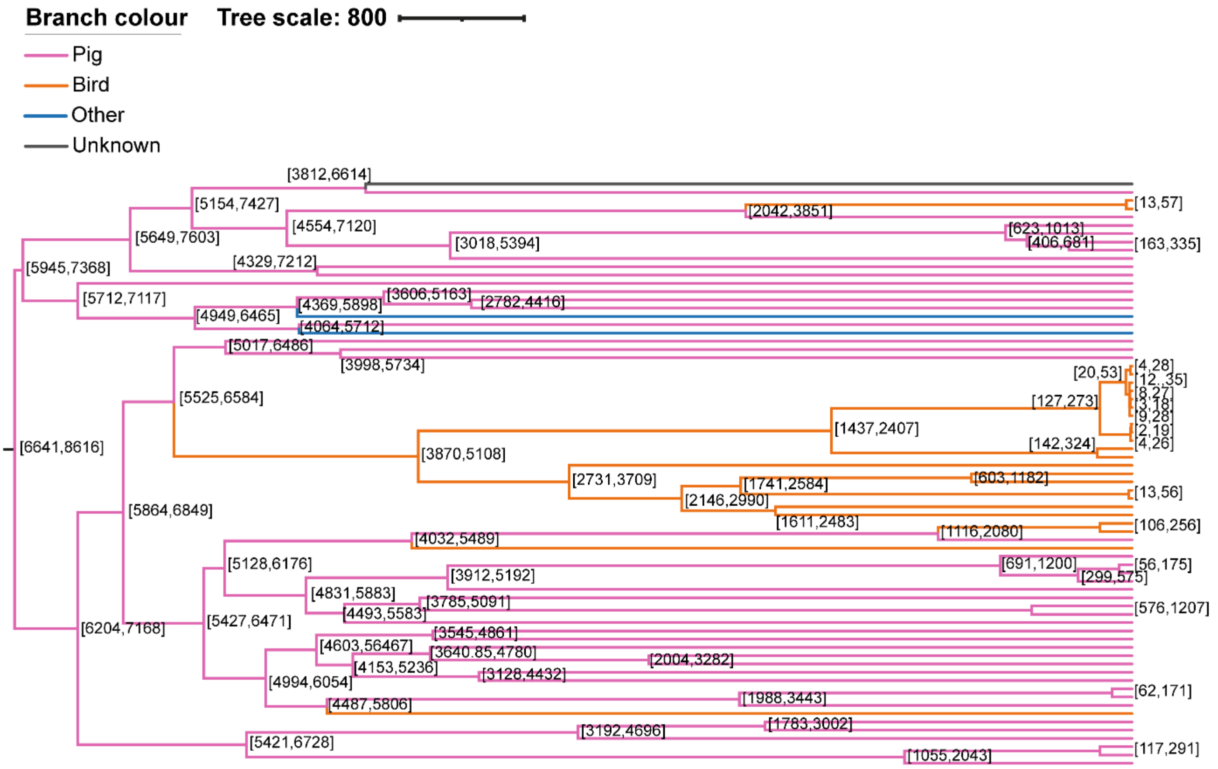

Figure S8. Height_95%_HPD confidence intervals of BEAST tree. Same tree as shown in Figure S7 but with height_95%_HPD confidence intervals and visualised in Figtree (45). Tree branches are coloured according to host species: pig (pink), bird (orange), other (blue), unknown (grey). The tree scale represents the number of years. Node labels indicate height_95%_HPD confidence intervals. The metadata of the tree is listed in Table S28.

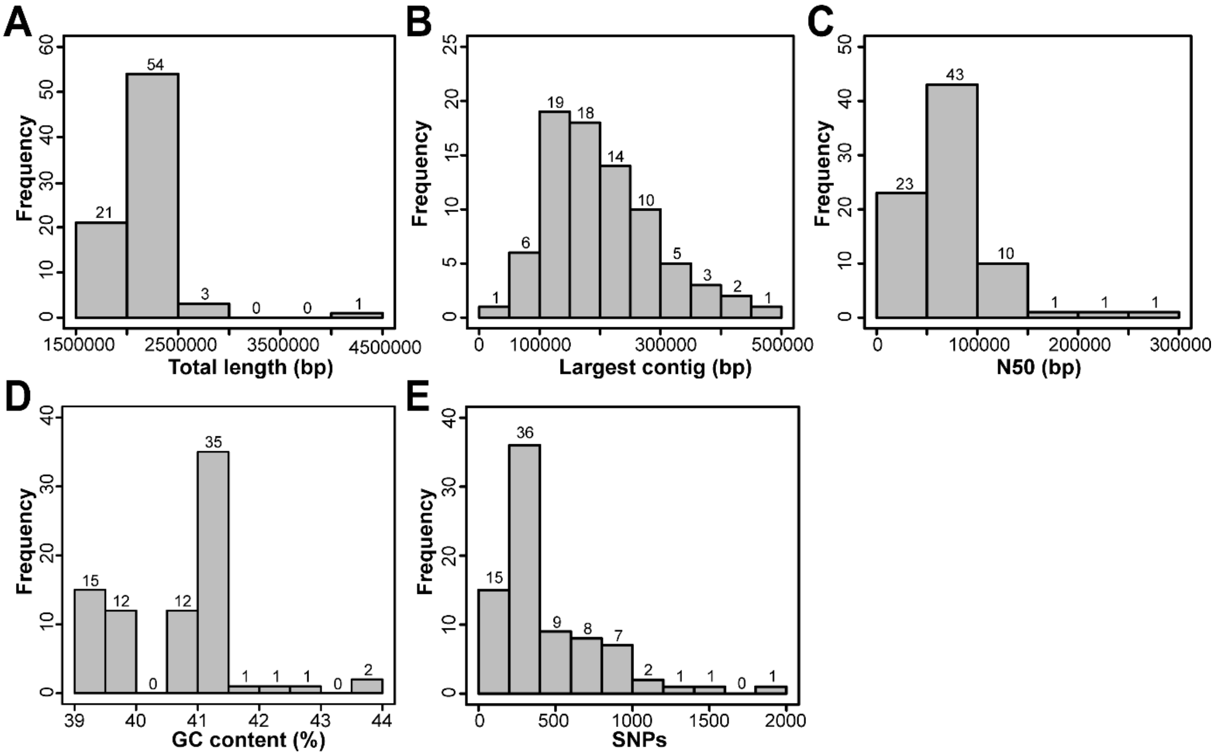

Figure S9. Quality control analysis with Quast. Quality control of all genome sequences from Berlin, Vietnam and other publicly available sequences was performed with Quast (63). (**A**) shows the total

length of the genome in base pairs (bp). In (**B**) the length of the larges contig is depicted, while (**C**) shows the N50 value. The GC content is displayed in (**D**) and normally ranges from 39 to 41% in *S. suis*. (**E**) shows the number of single nucleotide polymorphisms (SNPs).

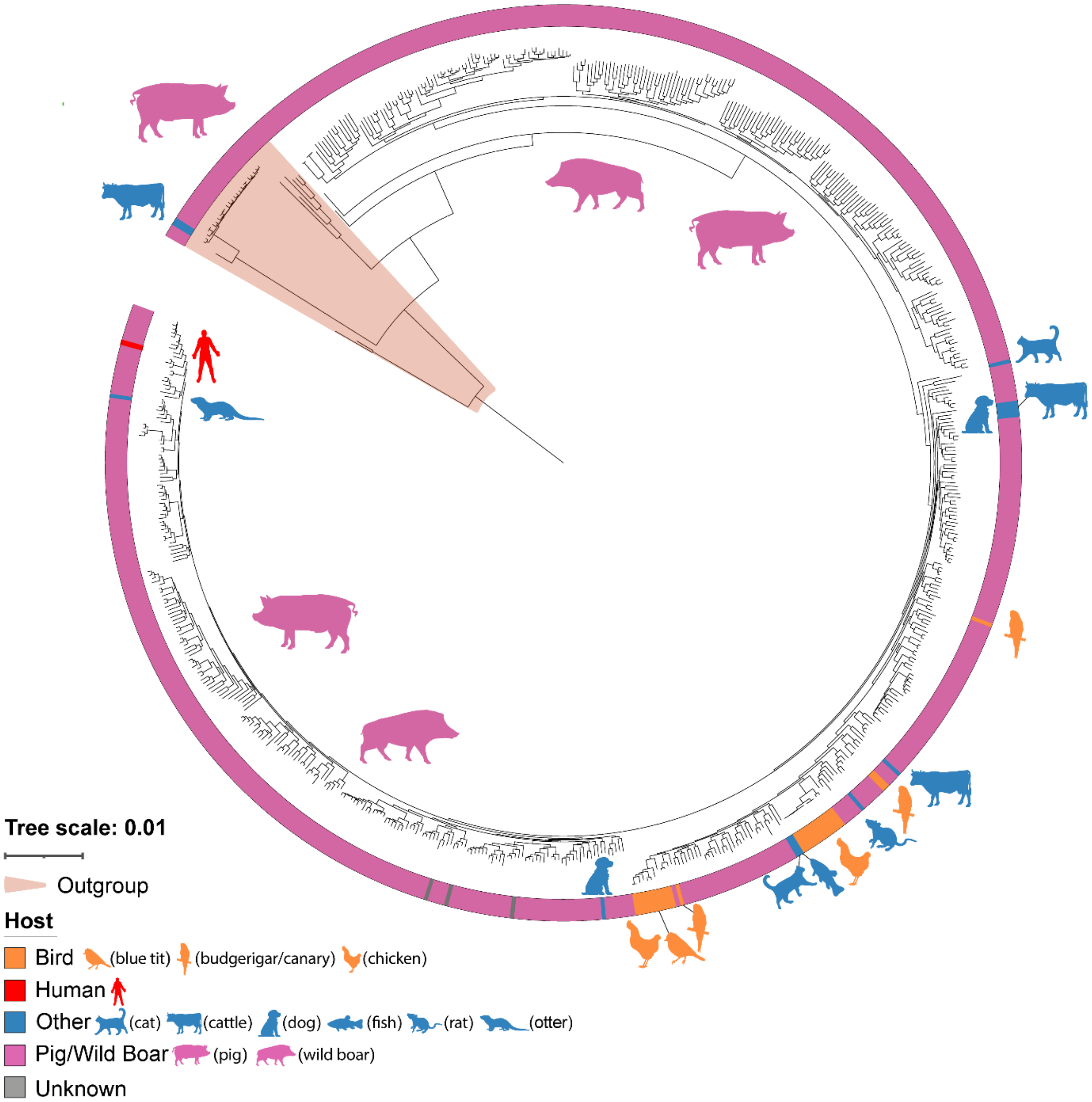

Figure S10. Thirty-two of the Vietnamese pig isolates and two cattle isolates form an outgroup in the phylogenetic tree. Prokka v.1.13.3 was used to annotate the genomes (52). Orthologous genes were identified, and alignments of the shared genes were created with Panaroo v.1.2.7 (53). The approximately-maximum-likelihood phylogenetic tree was created using Fasttree and visualized with ITOL v.6.7.3 (54, 55). The tree includes one *S. suis* isolate of each lineage identified in Murray et al. (16), the Vietnamese pig isolates and the *S. suis* isolates from birds and other species (23, 25, 26). The tree scale represents the number of substitutions per site. The host species is indicated in the outer ring. Silhouettes highlight the different host species. Most of the Vietnamese pig isolates as well as the isolates IMT53434_Cattle and IMT54075_Cattle are very distinct from the rest of the population and were therefore excluded from further analysis (red coloured background). Only seven Vietnamese pig isolates remain in the dataset: 3518_Vietnamese_Pig, 3519_Vietnamese_Pig, 3521_Vietnamese_Pig, 3523_Vietnamese_Pig, 3524_Vietnamese_Pig, 3526_Vietnamese_Pig and 3530_Vietnamese_Pig.

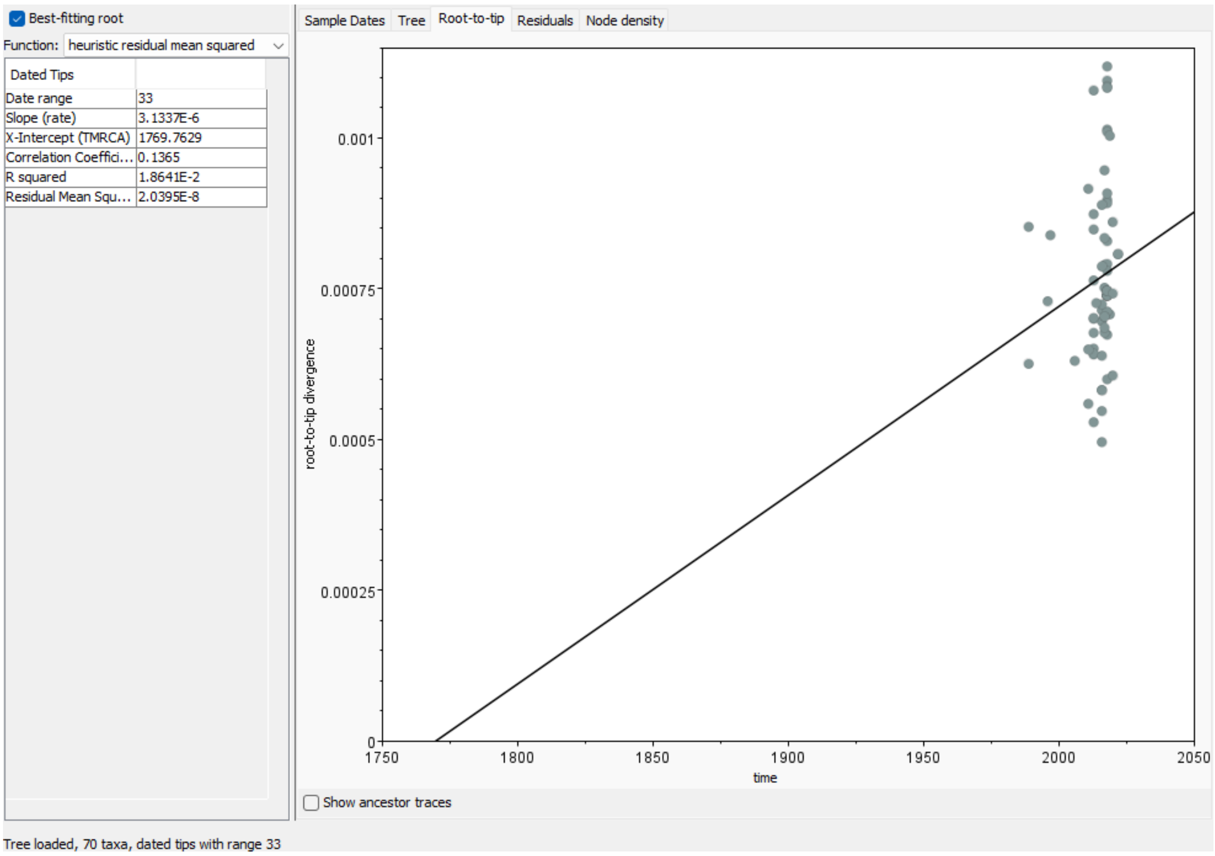

Figure S11. No temporal signal detected with TempEst. To investigate the molecular epidemiology of the bird lineage, we produced a reference-mapped assembly of the pruned tree group with Bowtie2 using the strain D12 as a reference (129, 140). Regions of recombination were identified using Gubbins v3.3.1 (93). Only non-recombined sites with less than 10% gaps were kept. Gaps were removed with the ape package in R (87). Temporal signal was analysed with TempEst software investigating the relation between genetic divergence over time and sample isolation dates (89). The strain D12 was excluded from this analysis as its isolation date is unknown. Rates calculated from the slope of distance from root over time were consistent with previous estimates of clock rates for *S. suis* (16). Root-to-tip graph is shown with a slope of 3.1337E -6 and an R squared value of 1.8641E-2.
